# Regulatory roles of miR-22/Redd1-mediated mitochondrial ROS and cellular autophagy in ionizing radiation-induced BMSC injury

**DOI:** 10.1038/s41419-019-1373-z

**Published:** 2019-03-07

**Authors:** Zhonglong Liu, Tao Li, Fengshuo Zhu, Si’nan Deng, Xiaoguang Li, Yue He

**Affiliations:** 1 Department of Oral Maxillofacial & Head and Neck Oncology, Shanghai Ninth People’s Hospital Affiliated to Shanghai Jiao Tong University School of Medicine, Shanghai, China 200011; 2 Department of Orthopedics, Shanghai Ninth People’s Hospital Affiliated to Shanghai Jiao Tong University School of Medicine, Shanghai, China 200011; 3 Department of Stomatology, Central Hospital of Min-Hang District, Shanghai, China 201109

## Abstract

Ionizing radiation (IR) response has been extensively investigated in BMSCs with an increasing consensus that this type of cells showed relative radiosensitivity in vitro analysis. However, the underlying mechanism of IR-induced injury of BMSCs has not been elucidated. In current study, the regulatory role of miR-22/Redd1 pathway-mediated mitochondrial reactive oxygen species (ROS) and cellular autophagy in IR-induced apoptosis of BMSCs was determined. IR facilitated the generation and accumulation of mitochondrial ROS, which promoted IR-induced apoptosis in BMSCs; meanwhile, cellular autophagy activated by IR hold a prohibitive role on the apoptosis program. The expression of miR-22 significantly increased in BMSCs after IR exposure within 24 h. Overexpression of miR-22 evidently accelerated IR-induced accumulation of mitochondrial ROS, whereas attenuated IR stimulated cellular autophagy, thus advancing cellular apoptosis. Furthermore, we verified Redd1 as a novel target for miR-22 in rat genome. Redd1 overexpression attenuated the regulatory role of miR-22 on mitochondrial ROS generation and alleviated the inhibitive role of miR-22 on cell autophagy activated by IR, thus protecting BMSCs from miR-22-mediated cell injury induced by IR exposure. These results confirmed the role of miR-22/Redd1 pathway in the regulation of IR-induced mitochondrial ROS and cellular autophagy, and subsequent cellular apoptosis.

## Introduction

Radiotherapy, known as ionizing radiation (IR), is widely performed as a major or adjuvant treatment for malignancies. This therapeutic method is substantially detrimental to cancer cells and simultaneously induces DNA damage, cell cycle arrest, apoptosis and destroys the metabolic balance of bony tissues^1,2^. Bone marrow mesenchymal stromal cells (BMSCs) are the most important cell type in bone marrow, as they provide osteogenic potential and regulate immunity and angiogenesis. The IR responses of BMSCs, including alterations of cell viability and differential capacity, have been widely investigated in the study of osteoradionecrosis pathogenesis, and there is an increasing consensus that these progenitor cells show relative radiosensitivity, characterized by increased apoptosis and prohibitive osteogenic capacity ratios both in vivo and in vitro^2–8^. Thus, knowledge of the mechanism underlying how BMSCs maintain their viability and protect cells from IR-induced injury is particularly critical in tissue renewal and subsequent regeneration.

IR exposure triggers the accumulation of reactive oxygen species (ROS) and persistent oxidative stress. Most ROS originate from the perturbation of mitochondrial metabolism that occurs in the electron transport chain (ETC), which disturbs energy production and the cellular redox status^9,10^. In addition, direct damage caused by IR leads to a dysfunctional mitochondrial status, thus impairing the antioxidative defense system and further promoting ROS accumulation^11^. Previous studies have demonstrated that mitochondrial ROS play roles in the contributions of TGF-β1, IR, butyrate, H_2_O_2_ and myocardial ischemia/reperfusion-induced cellular apoptosis^9,11–15^. This mitochondrial apoptosis pathway is initiated by ROS stimulation, followed by mtDNA damage, impaired antioxidant defense and loss of mitochondrial membrane potential (MMP)^16^. However, the mediator role of mitochondrial ROS in IR-induced BMSC injury and the molecular mechanism are still unclear.

Recent studies have also shown that the resistance of malignancies to radiotherapy is associated with the activation of cellular autophagy^17^. IR-induced autophagy exerts cytoprotective functions by eliminating dangerous signals, including ROS, inflammation and metabolic precursors, and alleviates mitochondrial damage^18^. Autophagy activated by IR prevents MSC injury and maintains stemness by decreasing intracellular ROS generation^19^. These results indicate that the viability of irradiated BMSCs can be preserved by inhibiting mitochondrial ROS and promoting cellular autophagy. However, the regulatory role that autophagy plays in IR-induced BMSC injury and the molecular mechanism also deserve more attention.

microRNA-22 (miR-22) belongs to a small non-coding RNA family and functions in the gene silencing and post-transcriptional regulation of mRNA. Growing evidence supports that miR-22 is involved in multiple cellular biological processes, including radiation, proliferation, apoptosis, ROS, autophagy, cell survival, neuroprotection, and myocardial ischemia/reperfusion injury^13,15,20–22^. However, the regulatory roles of miR-22 in IR-induced mitochondrial ROS, cellular autophagy and the subsequent apoptosis have not been elucidated. Using TargetScan prediction, we found that Redd1 (also called DDIT4) is directly targeted by miR-22. This mRNA could be induced by radiation and participates in the regulation of DNA damage, ROS, autophagy and apoptosis^23,24^. In γ-radiation cell model, Li et al. found that Redd1 was negatively regulated by miR-30c evidenced by that overexpression of miR-30c suppressed Redd1 level resulting in human fetal osteoblast cell death^25^. This finding indicated an intimate association of microRNA with Redd1 in biological processes.

As mentioned above, we postulated the hypothesis that miR-22/Redd1 pathway-mediated mitochondrial ROS and cellular autophagy may play pivotal regulatory roles in IR-induced BMSC injuries. The analysis described herein may further probe the molecular mechanism regulating IR-induced apoptosis.

## Materials and methods

### Isolation and in vitro culturing of rBMSCs

Male Sprague–Dawley rats (4 weeks old) were obtained from the Experimental Animal Center at our institution. After being immersed in 75% ethanol for 10 min, the bilateral tibias of the rats were dissected from the surrounding tissues. The bone marrow was then repeatedly flushed with a 1 ml syringe (BD Biosciences, San Jose, CA, USA) into a dish filled with culture medium. After centrifugation at 1000 r.p.m. for 5 min, the cellular sediment was resuspended in complete medium containing 10% FBS (Gibco, Thermo Fisher Scientific, MA, USA), α-MEM (HyClone, USA), and 1% penicillin-streptomycin (HyClone). The suspension was filtered with a 70-μm cell strainer (BD Biosciences) and then seeded into a 25 cm^2^ flask. The cell medium was changed every 2 days, and the cells were expanded at a 1:3 ratio upon reaching 80–90% confluence.

### Irradiation procedure

Rat BMSCs (rBMSCs) of the fourth passage were cultured in 6-cm dishes and moved to the radiotherapy room when the cells reached 80% confluence. The cells were subjected to IR (electrons) using 6 MeV (Precise Treatment System, Elekta, Sweden) at a dosage of 6 Gy and a dose rate of 600 Mu. The cells were then placed back in the incubator for continuous culturing before the samples were collected for further analysis.

### Measurement of total intracellular ROS levels

rBMSCs were seeded in 12-well plates in triplicate at a density of 2 × 10^4^ cells/well. After routine culturing (with or without N-acetylcysteine, NAC: 5 mM) or miR-22 transfection, the cells were exposed to 0 or 6 Gy of IR, and analysis was performed at 24 h after X-ray exposure. For ROS staining, the cells were incubated with the Fluorometric Intracellular ROS Kit (Sigma-Aldrich, Merck, Germany) for 45 min (5% CO2, 37 ℃), gently washed with PBS 3 times, counterstained with 10 μg/ml Hoechst (Sigma-Aldrich, Merck) for 10 min, and then imaged under a fluorescence microscope. To determine the fluorescence intensity, the incubated cells were measured on a flow cytometer with the following parameters: *λ* ex = 640 nm and *λ* em = 675 nm. The results are shown as images and fluorescence intensity.

### MitoTracker^®^ Green FM and MitoSOX^TM^ Red staining

After pretreatment with MitoQ (5 µM) or gene modification followed by IR exposure, rBMSCs growing in 12-well plates were incubated with MitoTracker^®^ Green FM (Molecular Probes, Invitrogen, USA) at a concentration of 100 nM for 30 min. MitoTracker^®^ Green FM can passively diffuse across the plasma membrane and accumulate in active mitochondria. These incubated cells were washed with PBS 3 times, stained with MitoSOX^TM^ Red (Molecular Probes, Invitrogen) at a concentration of 5 μM diluted in Hank’s Balanced Salt Solution (HBSS)/Ca^2+^/Mg^2+^ for 10 min and then counterstained with Hoechst for an additional 10 min. The samples were then imaged under a fluorescence microscope using filters for FITC, PE and DAPI. The fluorescence intensities of MitoTracker^®^ Green FM and MitoSOX^TM^ Red were detected at 490/516 nm and 510/580 nm, respectively. MitoTracker Green FM was used as an endogenous reference for mitochondrial amounts in rBMSCs, and MitoSox Red was used as the counterstain to indicate mitochondrial ROS accumulation; the ratio of MitoSox Red to MitoTracker Green FM determined the final mitochondrial ROS level.Finally, the intensity ratio was calculated and presented as the mean ± S.E.M.

### Western blot analysis

Whole cell lysates were acquired using RIPA lysis buffer and phenylmethylsulfonyl fluoride (PMSF, 1 mM, Beyotime, China) by incubation on ice for 30 min. Protein concentrations were determined using the BCA Protein Assay Kit (PierceTM, Thermo Fisher Scientific). Equal amounts (25 μg/well) of protein samples were separated by SDS-PAGE (10%, 15%) and then transferred to polyvinylidene difluoride (PVDF, 0.45 or 0.22 μm) membranes (Millipore Corporation, MA, USA). The membranes were blocked with 5% BSA containing TBST for 1.5 h and then incubated with the following primary antibodies: anti-LC3 (2 μg/ml, Abcam), anti-Atg7 (1:500, Proteintech), anti-Atg12 (1:500, Santa Cruz), anti-Bcl-xl (1:1000, CST), anti-Bak (1:500, Santa Cruz), anti-Caspase-9 (1:500, Proteintech), anti-Bax (1:1000, Santa Cruz), anti-Cytochrome C (1:500, Proteintech), anti-Redd1 (1:1000, Proteintech), anti-Phospho-p70 S6 Kinase (Thr389) (1:1000), and anti-GAPDH (1:5000, Bioworld Technology, Inc, USA). After washing 3 times with TBST, the membranes were further incubated with horseradish peroxidase (HRP)-tagged secondary antibodies for 1 h at room temperature. Finally, the protein bands were visualized by Odyssey V3.0 image scanning (LI-COR, Lincoln, NE, USA). The densitometric intensities of the individual protein bands were quantified using Photoshop software, and the values of each sample were normalized to those of GAPDH.

### Ad-mCherry-LC3B transfection and autophagy analysis

rBMSCs cultured on 12-well plates were pretreated with an autophagy agonist (rapamycin) and autophagy inhibitors (3-MA and MHY1485) for 12 h, and miR-22 or Redd1 genetic modifications occurred for 24 h. The pretreated cells were transfected with Ad-mCherry-LC3B (Beyotime), which expresses the mCherry-LC-3B protein, at a multiplicity of infection (MOI) of 50 for 24 h. Subsequently, the transfected cells were exposed to 6 Gy of IR and further counterstained with Hoechst. The autophagy status and Hoechst staining were then observed and captured 24 h post-IR with fluorescence microscopy using filters for PE and DAPI, respectively. The autophagy level was assessed by the presence and number of red spots.

### JC-1 staining to detect mitochondrial membrane potential depolarization

rBMSCs cultured on 12-well plates were pretreated with MitoQ or modified with miR-22 and then subjected to 0 or 6 Gy of radiation. The samples were then incubated with JC-1 Mitochondrial Membrane Potential Dye (eBioscience^TM^, Invitrogen, Thermo Fisher Scientific) at a concentration of 5 μg/ml for 20 min at 37 °C. After rinsing with PBS 3 times, the cells were counterstained with 10 μg/ml Hoechst (Sigma-Aldrich, Merck) for 10 min. To observe MMP depolarization (initiation of apoptosis), we captured images of different samples under a fluorescence microscope using the FITC filter.

### Caspase-3 activity analysis

rBMSCs seeded on 6-cm plates were pretreated with NAC, an autophagy agonist, autophagy inhibitors, miRNA modification or mRNA modification and then exposed to 6 Gy of radiation. Caspase-3 activity was measured according to specifications of the Caspase-3 Activity Assay Kit (Beyotime). Briefly, the detection samples were acquired by cell lysis and centrifugation at 4 °C. This assay was based on the principle that Ac-DEVD-pNA (acetyl-Asp-Glu-Val-Asp p-nitroanilide) is catalyzed by caspase-3 and then produces pNA (*p*-nitroaniline), which gives a yellow color. We read the absorbance of each sample at 405 nm and calculated the caspase-3 activity in combination with the standard curve and protein concentration. The results are presented as the mean μM/mg prot ± S.E.M.

### miRNA isolation, transcription and real-time PCR

Total miRNA was extracted using the miRcute miRNA Isolation Kit (TIANGEN Biotech, Beijing, China), and total miRNA was reverse transcribed using the miRcute miRNA First-Strand cDNA Synthesis Kit (TIANGEN Biotech). Briefly, poly(A) was added to the 3′ end of the miRNA, and this product was reverse transcribed using the oligo(dT) universal tag to produce first-strand cDNA. The relative miR-22 (Forward primer: 5′-acgcgAAGCTGCCAGTTGAAGAACTGT-3′) gene expression level was analyzed using the miRcute miRNA qPCR Detection Kit (SYBR Green, TIANGEN Biotech) on the 7300 Real-Time PCR system. U6 served as the endogenous normalization control, and the fold change in miR-22 expression was determined by the comparative CT method (2^−ΔΔCT^). Redd1 primer: forward- 5′-GTTGGCATCAGTTCGCTCAC-3′; reverse- 5′-AGGACGCTGGTTGATGAGGT-3′.

### In vitro Cell transfection

The miR-22 mimics, inhibitor, NC were designed and subsequently synthesized by GenePharma Corporation (Shanghai, China). The fragments of Redd1 were list as following: (1) siRNA NC, sense: 5′-UUCUUCGAACGUGUCACGUTT-3′, antisense: 5′-ACGUGACACGUUCGGAGAATT-3’; (2) siRNA1: sense: 5′-GCAAGAGCUGCCAUAGUGUTT-3′, antisense: 5′-ACACUAUGGCAGCUCUUGCTT-3′; (3) siRNA2: sense: 5′-GCCUGUUGAGUUCUGCCAATT-3′, antisense: 5’-UUGGCAGAACUCAACAGGCTT-3′; 4)siRNA3: sense: 5′-GCUGCUCAUUGAAGAGUGUTT-3′, antisense: 5′-ACACUCUUCAAUGAGCAGCTT-3′. Transfection was performed by using Lipofectamin 3000 and Opti-MEM, and the final concentrations for miR-22 mimics was 50 MOI, and others (inhibitor and siRNAs) were 100 MOI. As for overexpression, we constructed plasmid by using pEX-1 vector with cloning site of EcoRI/BamHI. Transfection procedure was applied according to the instruction from GenePharma Corporation. The transfected efficiency was evaluated by using RT-PCR analysis.

### Dual luciferase reporter assay

To predict the target mRNAs of rno-miR-22, the TargetScan, miRDB and microRNA.org databases were searched, and Redd1 was determined to directly associate with rno-miR-22. Two possible seed locations existed between Redd1 and rno-miR-22 (345-351, 525-532). The wild-type (WT) fragment of the Redd1 (NM_080906.2) 3′-UTR containing the miR-22 binding site (525-532) as well as its ‘seed’ mutant and PC (rno-miR-22 inhibitor sponge) sequences were synthesized in vitro by GenePharma (Shanghai, China). The miR-22 (sense: 5′-AAGCUGCCAGUUGAAGAACUGU-3′, antisense: 5′-AGUUCUUCAACUGGCAGCUUUU-3′) and miR-NC (sense: 5′-UUCUCCGAACGUGUCACGUTT-3′, antisense: 5′-ACGUGACACGUUCGGAGAATT-3′) fragments were also designed and synthesized by GenePharma. The Redd1 fragments were then cloned into the pmirGLO Dual-Luciferase miRNA Target Expression Vector (Promega). Using the Lipo3000 Reagent (Invitrogen), 293T cells were co-transfected with the pmirGLO vector containing either the WT, mutant or PC sequence along with miR-22 or miR-NC. Cell lysates were harvested at 24 h and 48 h after transfection, and luciferase activities were determined using the Dual Luciferase Reporter System (Promega) and a microplate reader (Tecan M1000, Switzerland).

### GSH/GSSG, mitochondrial SOD activity analysis

GSH, an intracellular antioxidant, reflects the scavenging capability of ROS, and GSSG, an oxidized form of GSH, indicates ROS accumulation. High GSH to GSSG ratios represent better oxidation resistance and vice versa. rBMSCs were seeded at a density of 2 × 10^5^ cells per 60-cm dish in triplicate, modified with miRNA or mRNA, and then exposed to 6 Gy of IR after 24 h. The detection samples were acquired 24 h post-IR by freezing/thawing and homogenization using the GSH and GSSG Assay Kit (Beyotime) and the CuZn/Mn-SOD Assay Kit with WST-8 (Beyotime), respectively, according to the manufacturers’ protocols. The reaction principle of GSH/GSSG analysis is as follows:

2GSH + DTNB------GSSG + 2TNB

NADPH + H^+^ + GSSG-----NADP^+^ + 2GSH

Merge: DTNB + H^+^ + NADPH-----2TNB + NADP^+^

The reaction principle of SOD activity analysis is as follows:
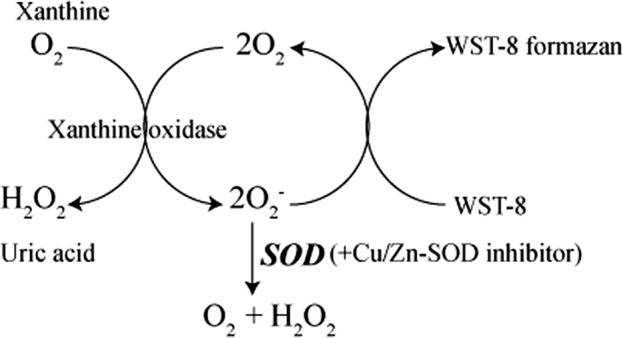


The mitochondrial SOD, i.e., Mn-SOD, was acquired by subtracting the CuZn-SOD from the total SOD. All experiments were repeated three times and presented as the mean ± S.E.M.

### Transmission electron microscopy analysis

rBMSCs subjected to different pretreatments were collected and subsequently fixed in 2.5% glutaraldehyde acid diluted in 0.1 M PBS buffer (pH 7.4) for 2 h at room temperature. The cells were scraped and suspended in fixation solution. The samples were dehydrated in acetone solution, embedded in Araldite (Basel, Switzerland), solidified, cut into 0.5-micron-thick sections, and then post-stained with uranyl acetate and lead citrate. A Philips CM-100 transmission electron microscope (TEM, FEI Company, Hillsboro, OR) was used to capture the autophagy images among the different samples.

### Statistical analysis

All data in the current study are presented as the Mean ± SD of 3 independent experiments. Data comparisons among different groups were performed using Student’s t tests or one-way analysis of variance (ANOVA) in SPSS (version 20, Chicago, IL, USA), and *p* ≤ 0.05 was deemed statistically significant.

## Results

### Radiation stimulated mitochondrial ROS and activated cellular autophagy

**Fig. 1:**
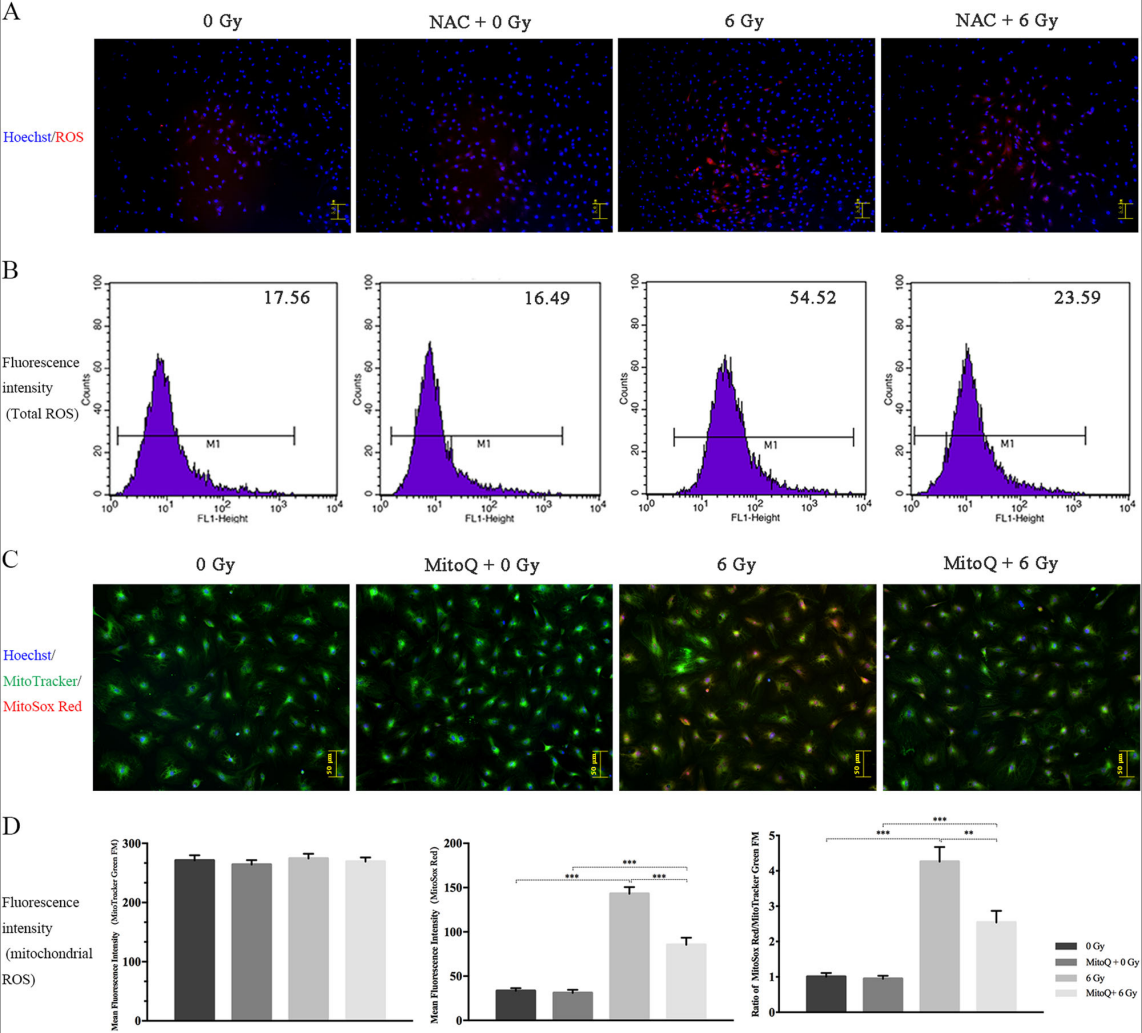
Induction role of ionizing radiation on ROS generation. **a** Intracellular total ROS staining and antioxygenation validation of NAC in BMSCs at 24 h post-IR. **b** Fluorescence intensity detection of intracellular ROS. **c** Mitochondrial ROS staining and antioxygenation validation of MitoQ in BMSCs at 24 h post-IR. **d** Ratio analysis of MitoSox Red/MtioTracker Green and validation of antioxygenation of MitoQ in BMSCs at 24 h post-IR. (**p ≤ 0.01; ***p ≤ 0.001)

Exposing rBMSCs to 6 Gy of X-ray radiation significantly induced total ROS production compared to that in the 0 Gy group when analyzed by both fluorescence microscopy and fluorescence intensity determined using fluorescence-activated cell sorting (FACS, 55.17 ± 1.93 vs 17.7 ± 0.958, *p* ≤ 0.001, Fig. 1a, b). Mitochondrial-dependent mechanisms are the major source of ROS in most cells. As expected, the mitochondrial ROS level in the 6 Gy group was significantly higher than that in the 0 Gy group (ratio: 4.26 vs. 1, *p* ≤ 0.001) (Fig. 1c, d). These data verified the inductive role of radiation in ROS generation. In addition, we observed the total and mitochondrial ROS in rBMSCs at different time points (6,12,24 and 48 h post-IR) with the peak at 24 h post-IR (Figure S1).

**Fig. 2:**
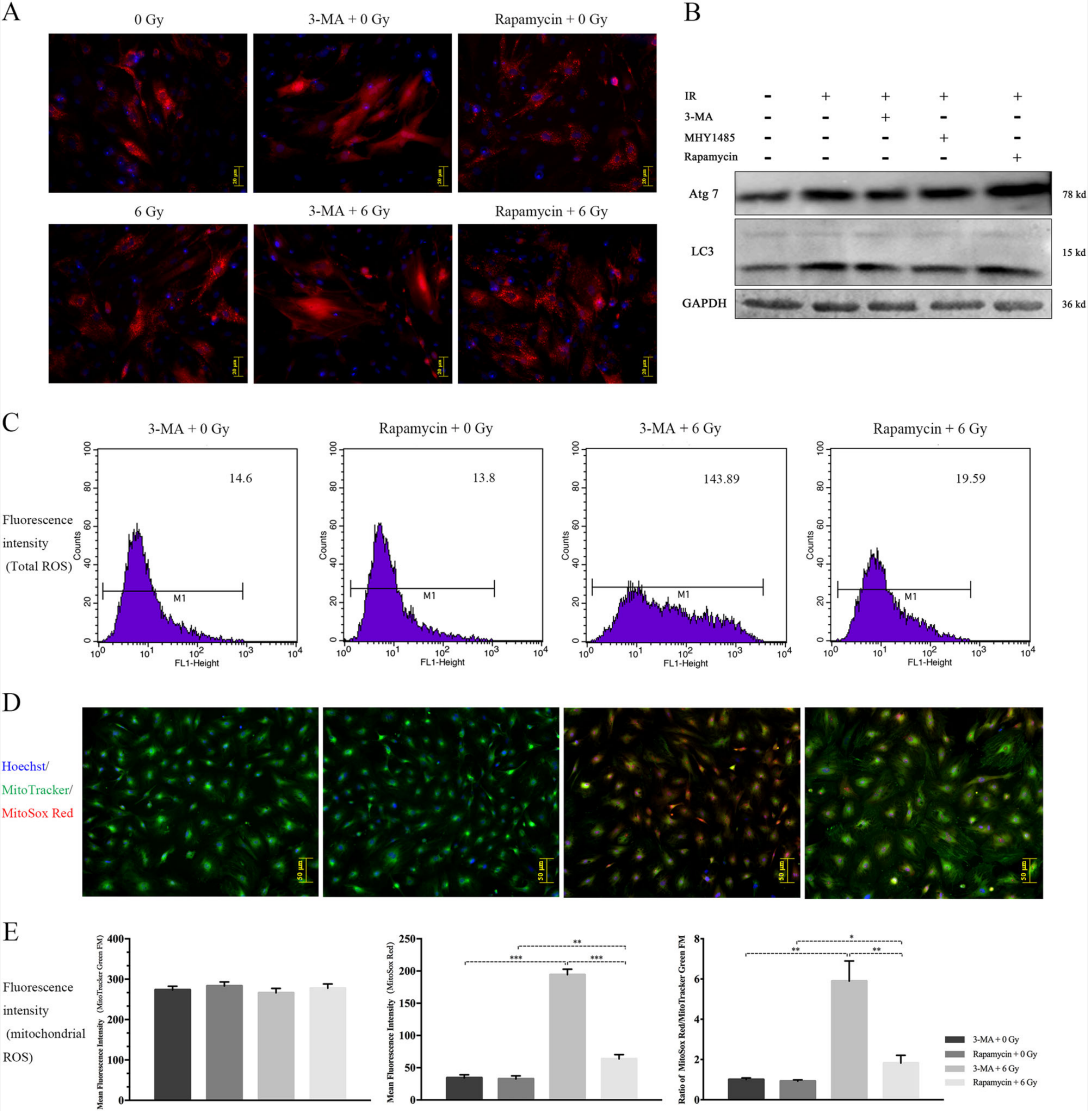
Activation role of IR on cellular autophagy and its regulatory effect on intracellular and mitochondrial ROS generation. **a** Analysis of Ad-mCherry-LC3B. **b** Protein expression of autophagy-related markers (LC3, Atg7) and interventive effect validation of autophagic agonist and inhibitor at 24 h post-IR. **c**, **d** Fluorescence intensity detection of intracellular ROS and mitochondrial ROS staining after Rapamycin and 3-MA intervention. **e** Ratio analysis of MitoSox Red/MitoTracker Green following autophagy intervention. (* ≤ 0.05; **p ≤ 0.01; ***p ≤ 0.001)

As a self-protective mechanism, autophagy was significantly triggered by IR exposure, demonstrated by the upregulation of LC-3 and Atg7 protein expression and the increased number of red spots in Ad-mCherry-LC3B staining (Fig. 2a, b). To further confirm autophagy activation during IR exposure, an autophagy agonist (rapamycin) and autophagy inhibitors (3-MA and MHY1485) were added prior to radiation. Rapamycin alone can stimulate cellular autophagy, and further improved IR-induced autophagy. In addition, 3-MA alone had no significant influence on the cellular autophagy in 0 Gy group, whereas remarkably inhibited IR-induced autophagy (Fig. 2a, b) (Figure S2).

In the correlation evaluation of mitochondrial ROS with cellular autophagy, we found that both total and mitochondrial ROS were obviously abated in the rapamycin/6 Gy group compared to those in the 3-MA/6 Gy group (Fig. 2c–e), indicating that IR-induced autophagy has a counteractive role in IR-stimulated mitochondrial ROS generation.

### Mitochondrial ROS and cellular autophagy play pivotal roles in the regulation of IR-induced BMSC apoptosis

**Fig. 3:**
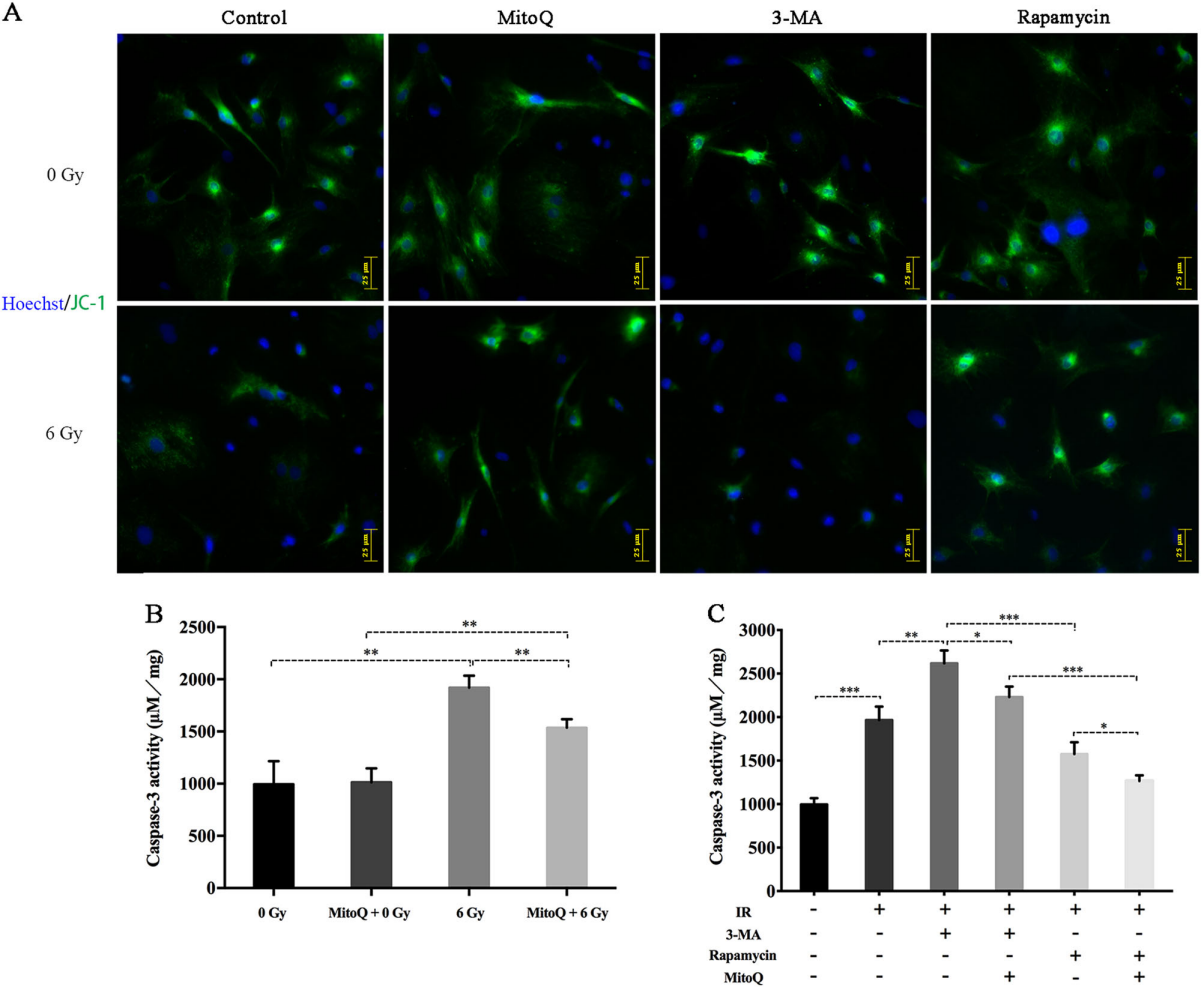
The regulatory role of radiation-induced mitochondrial ROS and autophagy on cellular apoptosis. **a** JC-1 analysis of mitochondrial membrane potetial at 24 h post-IR. **b** Caspase-3 activity detection following MitoQ intervetion and sebsequent IR exposure. **c** Caspase-3 activity detection following autophagic intervetion and sebsequent IR exposure. (*p ≤ 0.05; **p ≤ 0.01; ***p ≤ 0.001)

Excessive ROS accumulation may result in MMP depolarization, which is an indicator of early apoptosis in cells. The cells failed to maintain the MMP when exposed to 6 Gy of IR (Fig. 3a), reflecting the dysfunctional state of mitochondria and indicating the initiation of apoptotic program. Analysis of caspase-3 activity was used to verify that 6 Gy of IR distinctly increased the rBMSC apoptotic ratio compared with that in the 0 Gy group (1919 ± 66.71 vs 994.7 ± 127.3, *p* ≤ 0.01) (Fig. 3b).

We attempted to verify the regulatory role of mitochondrial ROS and autophagy in IR-induced apoptosis. MitoQ and NAC, antioxidants, have been proven to partially abolish mitochondrial and intracellular ROS accumulation, respectively. Pretreatment with NAC and MitoQ significantly reduced IR-induced intracellular ROS (NAC/6 Gy vs. 6 Gy = 23.82 ± 0.85 vs. 55.17 ± 1.93, *p* ≤ 0.001) and mitochondrial ROS (ratio of MitoQ/6 Gy to 6 Gy = 2.45:4.36, *p* ≤ 0.01) (Fig. 1a–d). Simultaneously, MitoQ also partially protected MMP depolarization and abated caspase-3 activity induced by 6 Gy of radiation (MitoQ/6 Gy vs 6 Gy = 1536 ± 46.4 vs 1919 ± 66.71, *p* ≤ 0.01) (Fig. 3a, b). These data verified that inhibiting ROS production induced by IR protects cellular viability and promotes lower apoptosis ratios.

Upon using autophagic interventions, we found that inhibiting autophagy significantly promoted IR-induced MMP loss and cellular apoptosis (6 Gy vs 3-MA/6 Gy: 1967 ± 89.69 vs 2543 ± 84.2, *p* ≤ 0.01), while promoting autophagy clearly protected MMP depolarization and reduced caspase-3 activity (Fig. 3c). An interesting finding was that pretreatment with MitoQ significantly reduced 3-MA promoted apoptosis induced by IR. This phenomenon supports that IR-activated autophagy plays a protective role in BMSCs against radiation injury, and this protective role may partially through inhibiting mitochondrial ROS genenration. In summary, mitochondrial ROS and autophagy play different roles, promoting and inhibiting the IR-induced rBMSC apoptotic program, respectively.

### Radiation-induced miR-22 expression and its role in IR-induced mitochondrial ROS and cellular autophagy

**Fig. 4:**
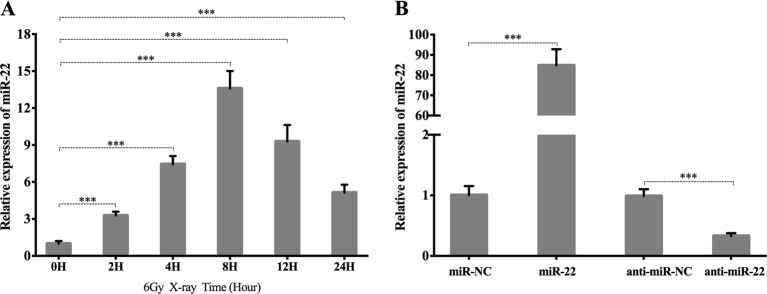
Radiation-induced expression of miR-22. **a** miR-22 expression at 0、2、4、8、12、24 h after 6 Gy radiation. **b** PCR verification of transfection efficiency of miR-22 mimics and inhibitor. (***p ≤ 0.001)

As demonstrated in Fig. 4a, the miR-22 level was significantly upregulated following IR exposure in a time-dependent manner, peaking at 8 h post-IR (13.6 ± 0.816-fold, *p* ≤ 0.001). MiR-22 expression was 84.8 ± 4.64-fold (*p* ≤ 0.001) and 0.33 ± 0.026-fold (*p* ≤ 0.01) higher than miR-NC and anti-miR-NC expression in cells transfected with a miR-22-mimic and a miR-22-inhibitor, respectively (Fig. 4b).

### miR-22 upregulation promotes IR-induced apoptosis by enhancing mitochondrial ROS and suppressing cellular autophagy

**Fig. 5:**
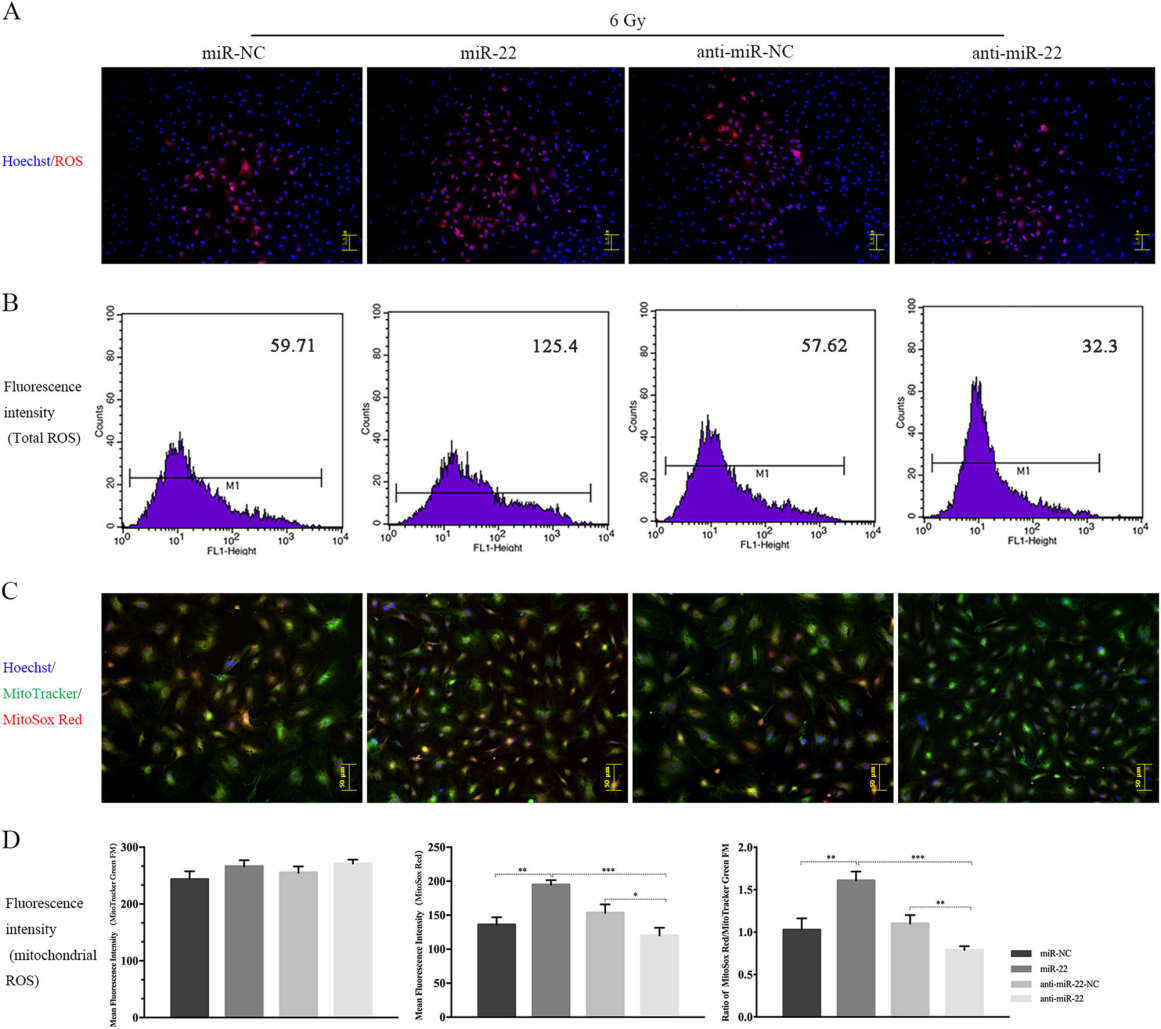
Regulatory role of miR-22 on the radiation-induced generation of ROS. **a** Intracellular total ROS staining in BMSCs following miR-22 modification and sebsequent IR. **b** Fluorescence intensity detection using flow cytometry. **c** Mitochondrial ROS staining in BMSCs following miR-22 modification and sebsequent IR. **d** Ratio analysis of MitoSox Red/MitoTracker Green. (*p ≤ 0.05; **p ≤ 0.01; ***p ≤ 0.001)

We next verified the functionality of miR-22 in IR-induced biological responses. Overexpression of miR-22 significantly aggravated, whereas downregulating miR-22 obviously attenuated IR-induced total ROS generation (126.5 ± 4.01 vs. 59.24 ± 2.26 (*p* ≤ 0.001) in the miR-22 and miR-NC groups and 31.9 ± 1.71 vs. 57.81 ± 3.47 (*p* ≤ 0.01) in the anti-miR-22 and anti-NC groups, respectively.(Fig. 5a, b). Mitochondrial ROS production also showed the same tendency as total ROS (Fig. 5c, d). We also found that miR-22 transfection alone (without IR exposure) had no significant effect on both total and mitochondrial ROS. (Figure S3)

**Fig. 6:**
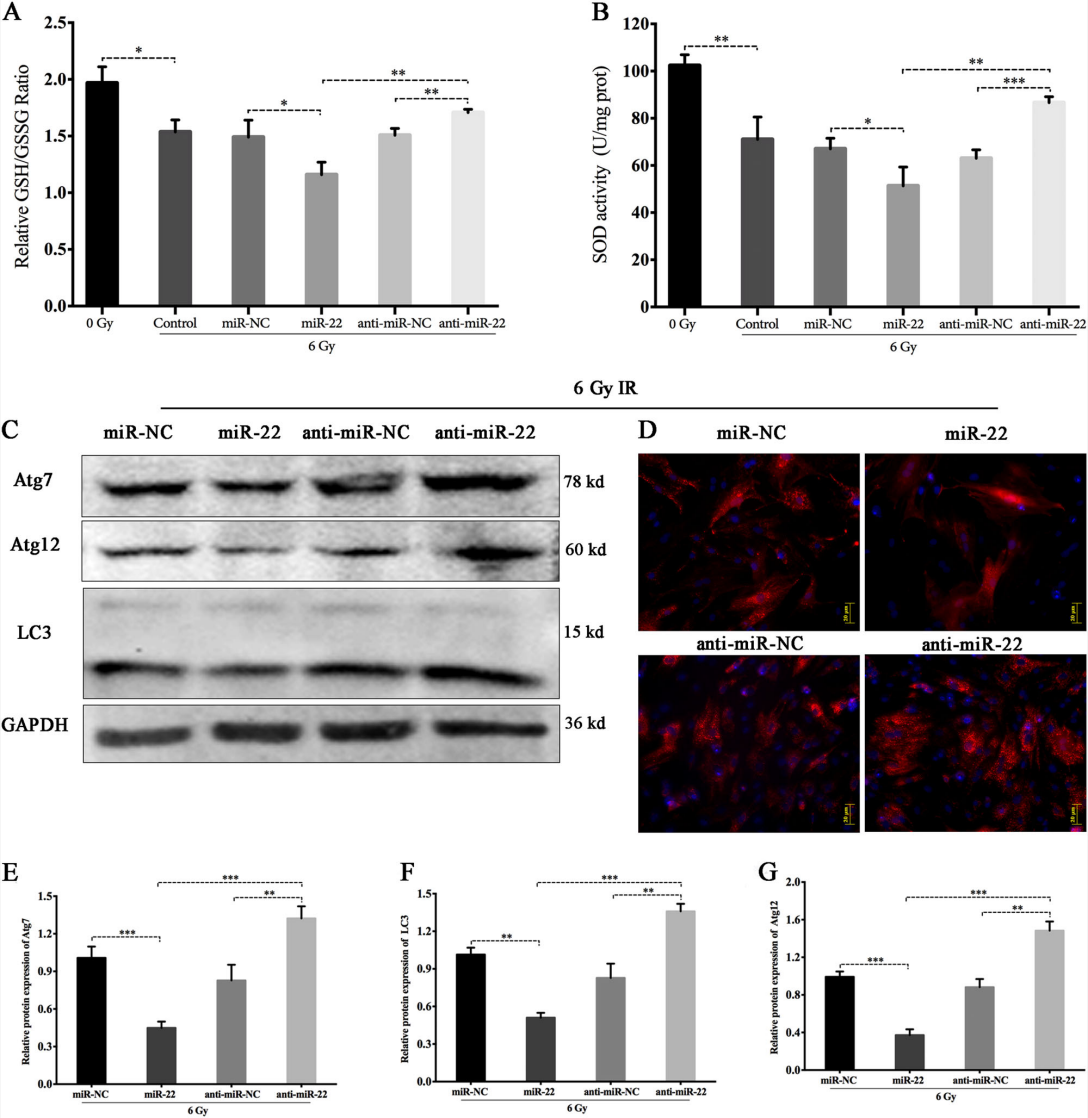
Regulatory role of miR-22 on the radiation-induced damage to intracellular antioxidant system and autophagy activation. **a** The ratio of GSH to GSSG in rBMSCs treated with miR-22 modification and IR exposure. **b** Mitochondrial SOD detection. **c** Protein expression of autophagy-related markers at 24 h after miR-22 modification and radiation. **d** Ad-mCherry-LC3B analysis. **e**–**g** Relative expression of autophagic related protein (Atg7, LC3, Atg12) under miR-22 modification and subsequent 6 Gy IR exposure. (*p ≤ 0.05; **p ≤ 0.01; ***p ≤ 0.001)

The antioxidant ability in the 6 Gy group was distinctly impaired compared to that in the 0 Gy group, characterized by a decreased GSH/GSSG ratio (1.97 ± 0.08 vs 1.53 ± 0.06, *p* ≤ 0.05, Fig. 6a). Furthermore, upregulating miR-22 clearly aggravated the impaired antioxidant capability, whereas inhibiting miR-22 significantly alleviated this impairment. The mitochondrial SOD activity showed a variation trend similar to that of GSH/GSSG (Fig. 6b).

For autophagy assessment, we detected the protein expression of LC-3, Atg7 and Atg12 following miR-22 genetic modification and subsequent IR. These protein levels were significantly attenuated by miR-22 overexpression and promoted by miR-22 downregulation (Fig. 6c, e–g). This regulatory role was further confirmed by Ad-cherry-LC3B immunofluorescence analysis, characterized by reduced and increased numbers of red spots in the miR-22 and anti-miR-22 groups, respectively (Fig. 6d).

**Fig. 7:**
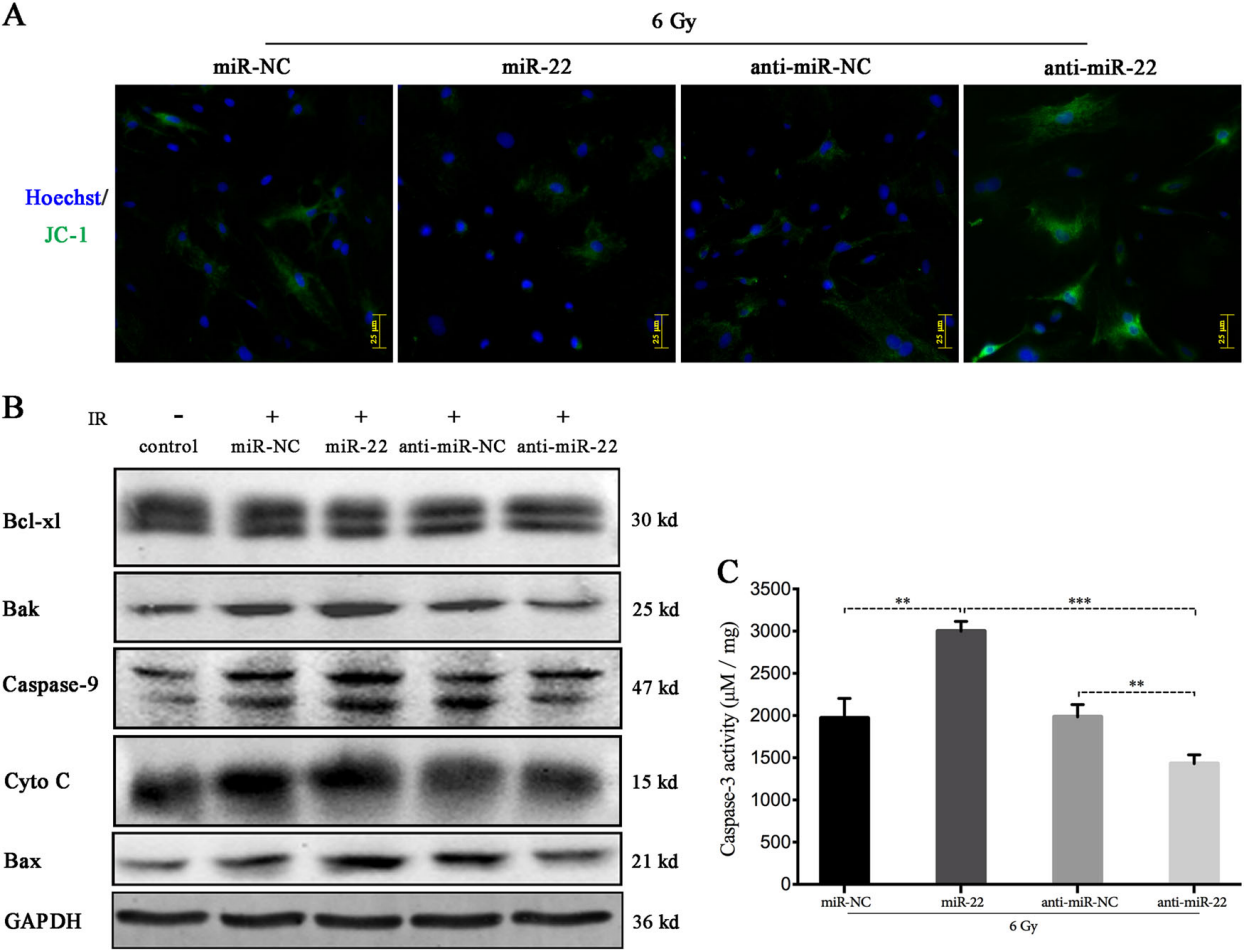
Regulatory role of miR-22 on the radiation-induced cellular apoptosis. **a** JC-1 analysis of mitochondrial membrane potetial at 24 h after miR-22 modification and radiation. **b** Mitochondrial-mediated apoptosis-related protein (Bcl-xl, Bak, Caspase-9, Cyto C, Bax) expression. **c** Caspase-3 activity analysis of rBMSCs following miR-22 transfection and subsequent IR. (**p ≤ 0.01; ***p ≤ 0.001)

We further verified whether miR-22 plays a regulatory role in IR-induced BMSC apoptosis. JC-1 staining indicated that miR-22 overexpression aggravated mitochondrial dysfunction, characterized by MMP depolarization (Fig. 7a). Subsequently, we attempted to verify the mechanism underlying mitochondrial-mediated cellular apoptosis by detecting apoptotic-related mitochondrial proteins. Cells transfected with miR-22 and then subjected to IR exhibited decreased Bcl-xl expression and increased Bak, caspase-9, cytochrome C, Bax expression compared to that in miR-NC-transfected cells. Inhibiting miR-22 increased Bcl-xl expression and decreased the expression of pro-apoptotic-related proteins (Fig. 7b, Figure S4).

Meanwhile, the analysis of caspase-3 activity showed that miR-22 aggravated IR-induced injury, and inhibiting miR-22 protected the viability of rBMSCs (Fig. 7c). We concluded that IR-induced rBMSC apoptosis is partially regulated by miR-22-mediated mitochondrial ROS accumulation and cellular autophagy inhibition.

### The 3’-UTR of Redd1 is a direct target of miR-22

**Fig. 8:**
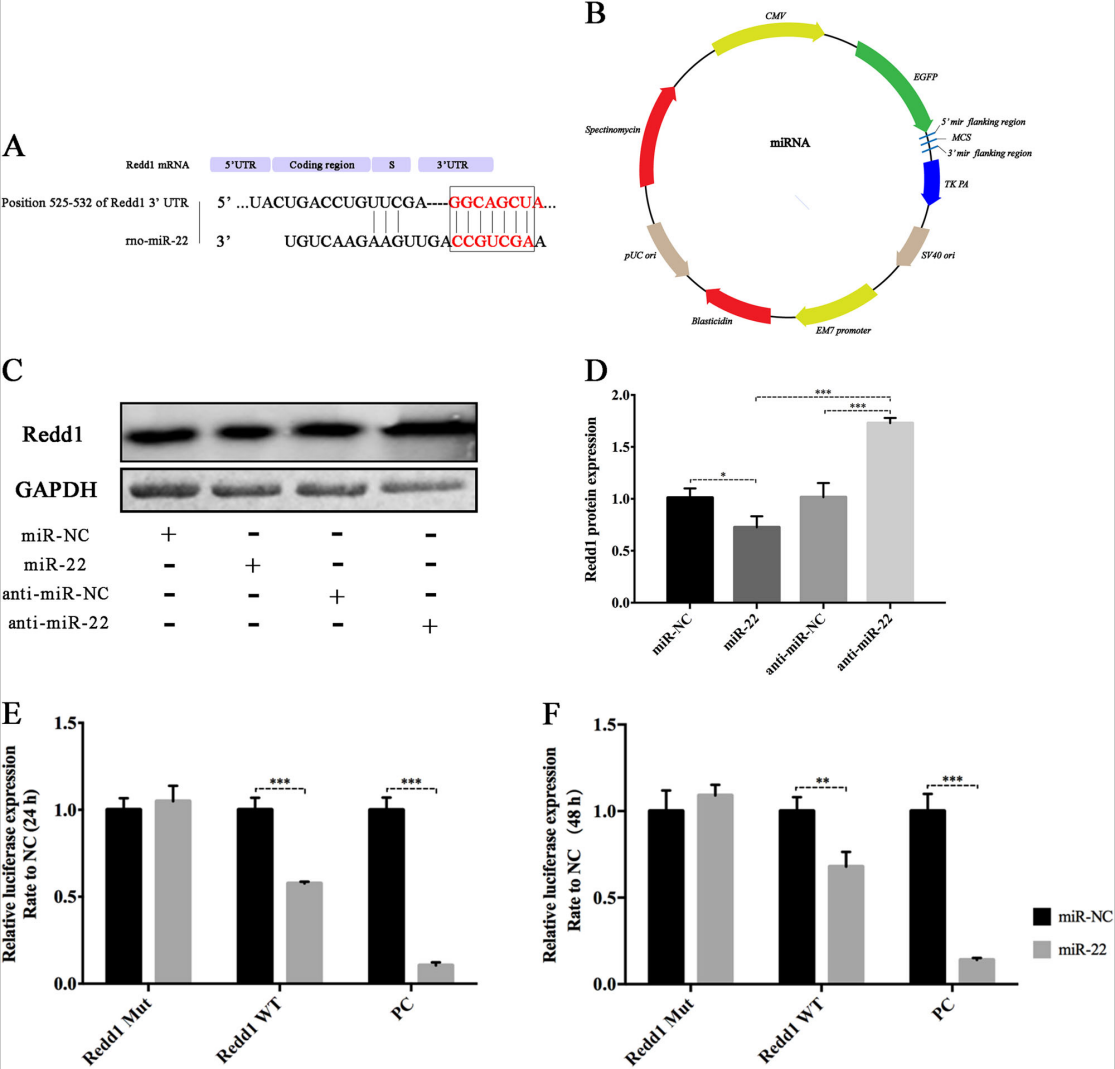
Dual luciferase report assay of miR-22 with Redd1. **a** Prediction of the binding site between miR-22 and Redd1. **b** miRNA vector construction. **c** The influnce of miR-22 on the protein expression of Redd1. **d** Quantitative analysis of protein expression. **e**, **f** Luciferase activity detection at 24 and 48 h following miR-22 and Redd1 transfection. (*p ≤ 0.05; **p ≤ 0.01; ***p ≤ 0.001)

After overlapping the prediction results from the TargetScan, miRDB and microRNA.org databases, Redd1, an important marker involved in the modulation of DNA damage, ROS and autophagy, was identified as a putative miR-22 target gene (Fig. 8a). Figure 8b shows the vector constructed for dual luciferase reporter analysis. Overexpression of miR-22 obviously suppressed Redd1 protein expression (0.72-fold, *p* ≤ 0.05), whereas silencing miR-22 significantly increased its protein expression (1.73-fold, *p* ≤ 0.001) in irradiated rBMSCs (Fig. 8c, d). To further confirm whether Redd1 is directly targeted by miR-22, we constructed WT and mutation (Mut) Redd1 fragments and inserted them into the pmirGLO vector together with miR-22 mimics or NC. We then transfected these constructs into 293 T cells, revealing a marked abatement of luciferase activity in cells co-transfected with Redd1 and miR-22 at both 24 (0.576-fold, *p* ≤ 0.001) and 48 h (0.679-fold, *p* ≤ 0.01) compared with that in cells co-transfected with the non-targeting miR-NC (Fig. 8e, f). These findings verified the existence of putative miR-22 binding sites located in the 3’ UTR of Redd1 mRNA.

**Fig. 9:**
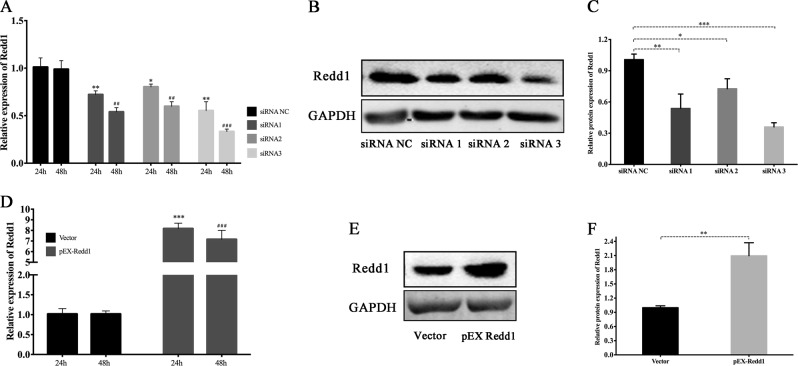
Verification of Redd1 genetic modification. **a** PCR analysis of transfection efficiency of siRNA. **b** Protein analysis of transfection efficiency of siRNA at 48 h post-transfection. **c** Relative protein expression of Redd1 following siRNA intervention. **d** PCR analysis of transfection efficiency of Redd1 overexpression. **e** Protein analysis of transfection efficiency of Redd1 overexpression at 48 h post-transfection. **f** Relative protein expression of Redd1 following overexpression intervention. (*p ≤ 0.05; **^/##^*p* ≤ 0.01; ***^/###^*p* ≤ 0.001)

To verify the functionality of Redd1 in IR-induced biological processes, we constructed Redd1 siRNA and overexpression vectors. The protein and gene expression of Redd1 showed the same trend (Fig. 9a–c). In addition, we successfully constructed an overexpression vector containing a Redd1 response element. Gene expression was upregulated by approximately 8.16-fold and 7.15-fold in rBMSCs at 24 and 48 h post-transfection, respectively (Fig. 9d–f).

### Upregulation of Redd1 attenuates the promotional effect of miR-22 on IR-induced injury by inhibiting mitochondrial ROS and promoting cellular autophagy

**Fig. 10:**
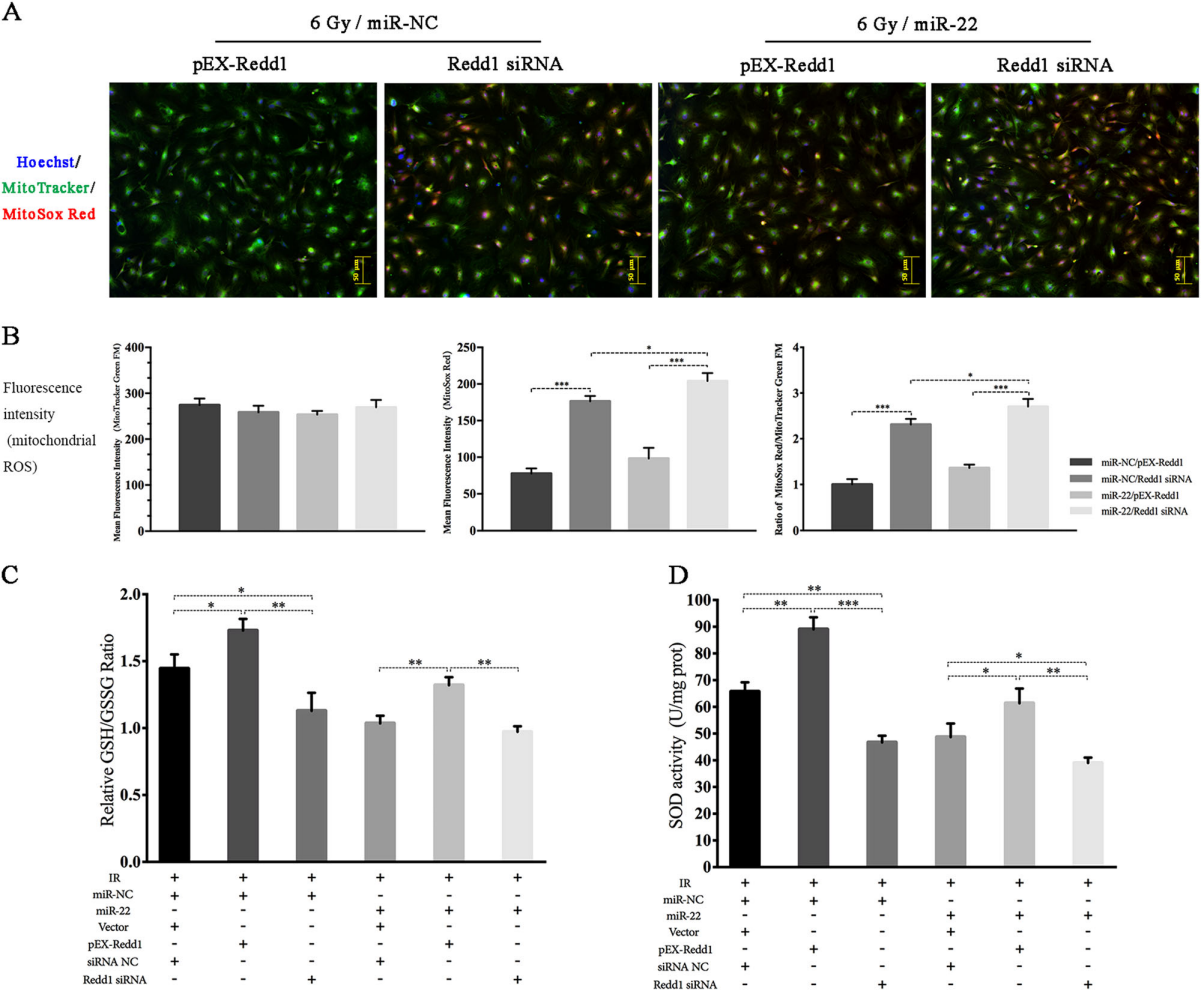
Regulatory role of miR-22/Redd1 on radiation-induced generation of mitochondrial ROS and damage to intracellular antioxidant system. **a** Mitochondrial ROS staining. **b** Ratio analysis of MitoSox Red/MitoTracker Green. **c**, **d** GSH/GSSG and mitochondrial SOD analysis in rBMSCs following miR-22, Redd1 modification and subsequent IR exposure. (*p ≤ 0.05; **p ≤ 0.01; ***p ≤ 0.001)

To determine whether Redd1 is involved in miR-22-mediated mitochondrial ROS accumulation and autophagy inhibition, we performed genetic modification experiments in rBMSCs with both Redd1 and miR-22. As shown in Fig. 10a–c, the upregulation of Redd1 attenuated mitochondrial ROS production, as evidenced by reduced mitochondrial ROS staining and an elevated GSH/GSSG ratio (miR-NC/pEX-Redd1/6 Gy vs miR-NC/6 Gy: 1.73 ± 0.049 vs. 1.44 ± 0.062, *p* ≤ 0.05), whereas silencing Redd1 significantly reduced this ratio. Furthermore, overexpression of Redd1 partially abolished the promotional effect of miR-22 on mitochondrial ROS induction, as proven by the increased GSH/GSSG ratio (1.32 ± 0.035 vs. 1.037 ± 0.032, *p* ≤ 0.01) compared to that in cells treated with miR-22/6 Gy alone. The regulatory role of Redd1 in the miR-22-mediated inhibition of SOD activity was consistent with that of GSH/GSSG (Fig. 10d). In summary, Redd1 overexpression attenuated IR-induced mitochondrial ROS generation while partially abating miR-22-mediated mitochondrial ROS induced by IR.

**Fig. 11:**
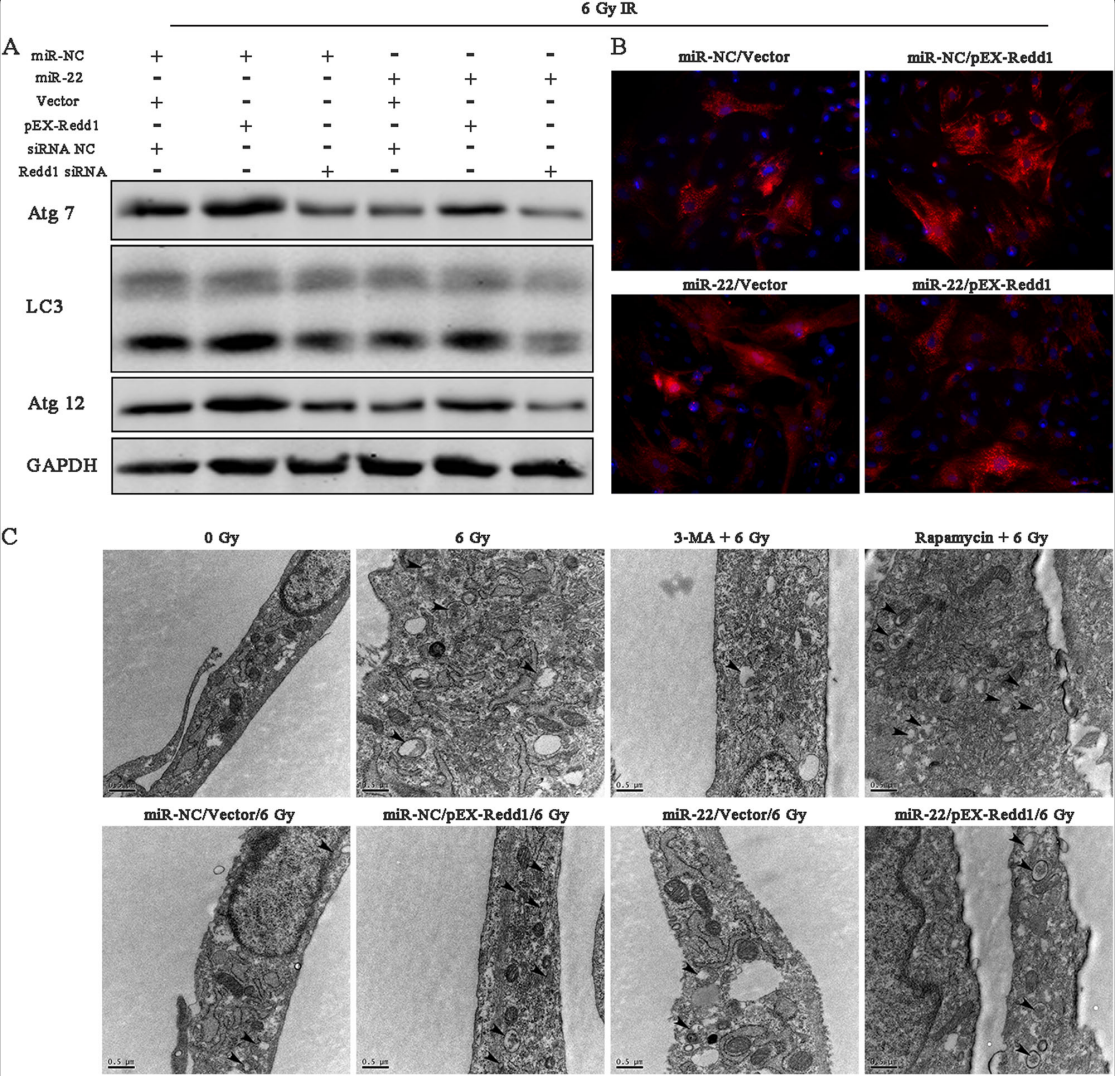
Regulatory role of miR-22/Redd1 on radiation-induced cellular autophagy. **a** Protein expression of autophagy-related markers at 24 h after Redd1/miR-22 modification and radiation. **b** Ad-mCherry-LC3B analysis. **c** (T.E.M analysis) Black arrow represents the autophagosome

As for autophagic regulation, upregulating Redd1 significantly increased IR-induced autophagy-related protein expression as well as the numbers of red spots and autophagosomes, whereas silencing Redd1 markedly reduced these values. In addition, Redd1 overexpression reversed miR-22-induced autophagy inhibition and vice versa (Fig. 11a–c, Figure S5). We further explored the mediation way of rapamycin on Redd1 expression and even miR-22 mediated suppression of autophagy. The findings indicated that rapamycin can promote the protein expression of Redd1, Atg7 and LC3 (Figure S6), meanwhile augment the number of autolysosome (Figure S7). All these demonstrated that rapamycin can be used as a cyto-protective liquid when encounter radiotherapy.

**Fig. 12:**
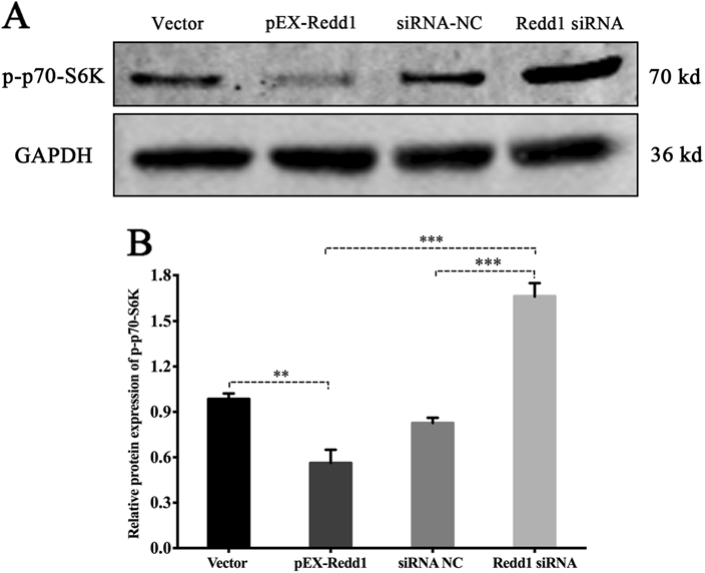
The negative regulation of Redd1 on mTORC1 activity. **a** Protein expression of mTORC1 downstream targets p70 S6 Kinase (p70-S6K). **b** Relative protein expression of p-p70-S6K. (**p ≤ 0.01; ***p ≤ 0.001)

Redd1 has been reported to participate in autophagy regulation through mTORC1pathway. To very this viewpoint, we detected the phosphorylation level of p70 S6 Kinase (p70-S6K) after 24 h of Redd1 modifications, with the finding that Redd1 overexpression significantly inhibited the phosphorylation of p70-S6K (mTORC1 inhibition), which indirectly reveal the negatively mediated role of Redd1 on mTORC1 status (Fig. 12). We can speculate that the Redd1-mediated effect on autophagy is mTORC1-dependent.

**Fig. 13:**
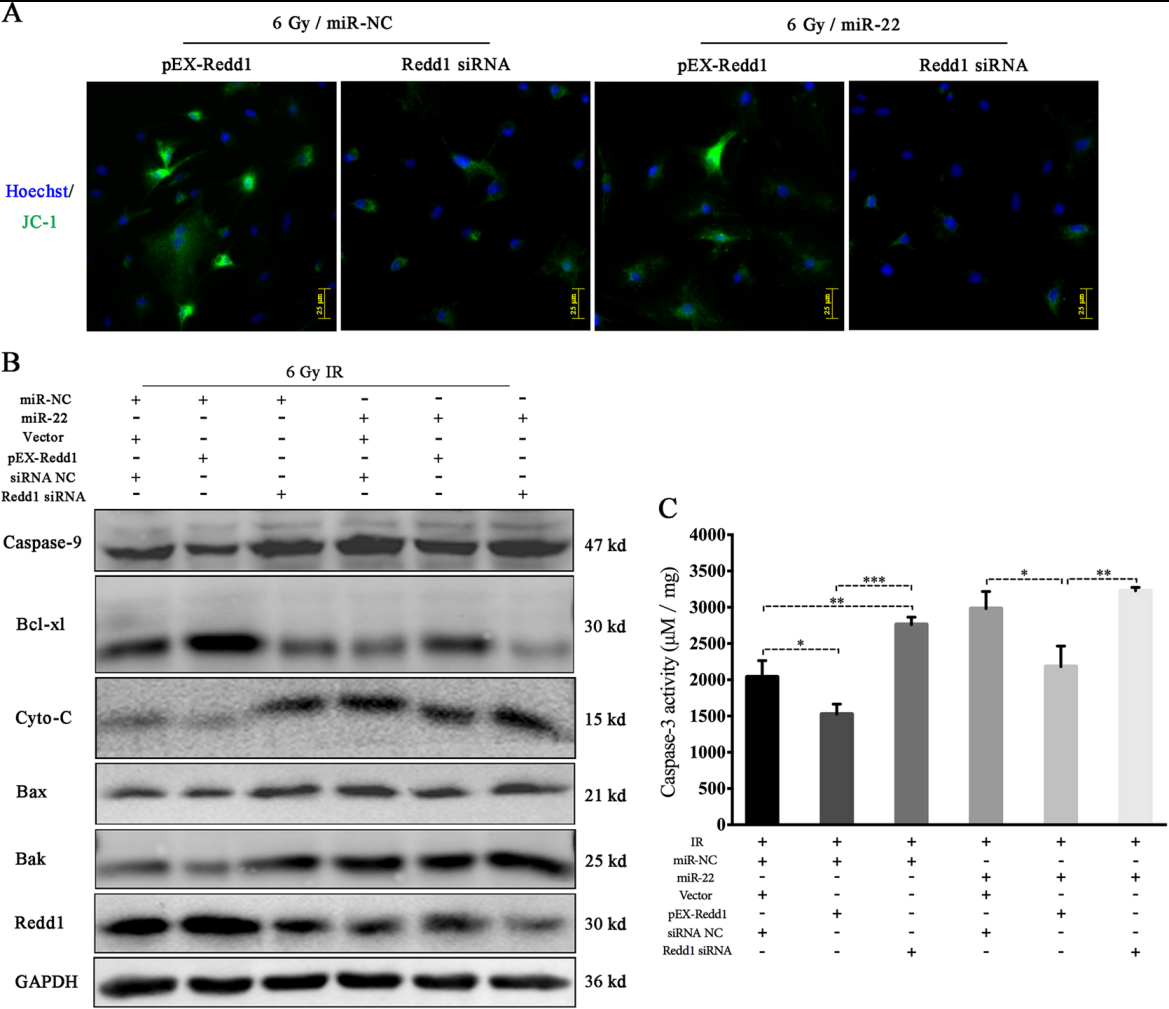
Regulatory role of miR-22/Redd1 on radiation-induced cellular apoptosis. **a** JC-1 analysis of mitochondrial membrane potetial at 24 h after genetic modification and subsequent radiation. **b** Mitochondrial-mediated apoptosis-related protein expression. **c** Caspase-3 activity analysis. (*p ≤ 0.05; **p ≤ 0.01; ***p ≤ 0.001)

Finally, we attempted to verify whether mitochondrial ROS and cellular autophagy mediated by the miR-22/Redd1 pathway plays a regulatory role in IR-induced rBMSC apoptosis. Overexpression of Redd1 protected rBMSCs from IR-induced apoptosis, characterized by MMP detection (Fig. 13a) and lower caspase-3 activity (miR-NC/pEX-Redd1/6 Gy vs miR-NC/6 Gy:1524 ± 80.56 vs. 2038 ± 131.5, *p* ≤ 0.05) compared to that in cells transfected with the non-targeting miR-NC. Silencing Redd1 had the opposite effect on IR-induced cell injury. In addition, co-transfection of miR-22 and Redd1 induced lower caspase-3 activity (miR-22/pEX-Redd1/6 Gy vs. mir-22/6 Gy: 2181 ± 163.6 vs. 2981 ± 136.6, *p* ≤ 0.05) compared to that in cells transfected with miR-22 alone, indicating that Redd1 overexpression might be a novel therapeutic for IR/miR-22-induced cell injuries (Fig. 13c). The role of Redd1 in mitochondrial-mediated cellular apoptosis was identical to that of caspase-3. (Fig. 13b, Figure S8).

**Fig. 14:**
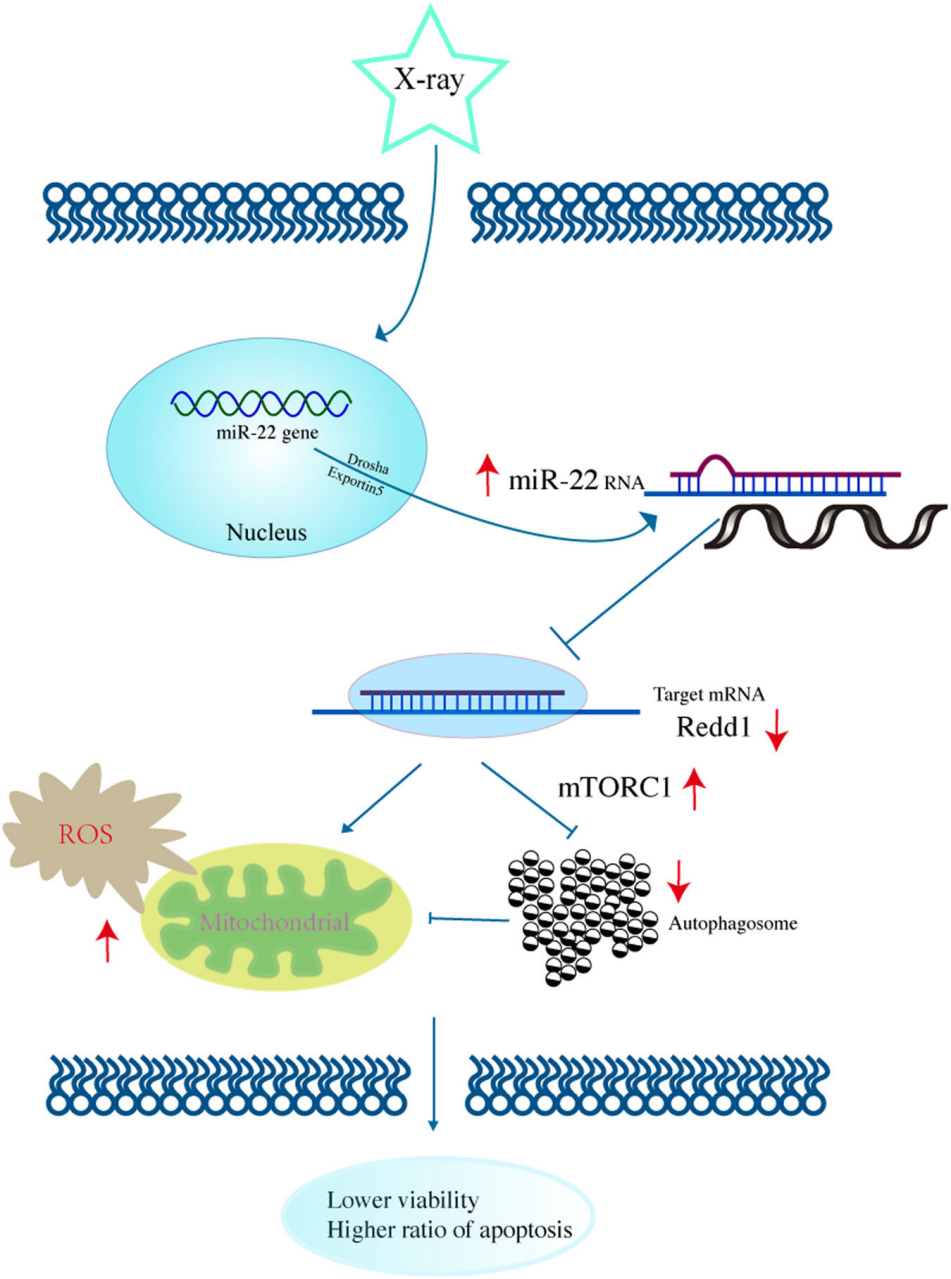
Molecular mechanism diagram

In conclusion, these data show that mitochondrial ROS and cellular autophagy are mediated by the miR-22/Redd1 signaling pathway, which plays a pivotal role in preserving cellular viability after IR exposure (Fig. 14).

## Discussion

Radiotherapy involving bony tissue always decreases BMSC viability, subsequently decreasing osteogenic inhibition. Accordingly, exploring the mechanism underlying IR-induced viability loss is essential. The IR dosage selected in the current study was determined by the cut-off point at which cell proliferation was not affected but showed increased cell apoptosis^19^. Apoptosis in BMSCs was significantly induced via exposure to 6 Gy of IR, characterized by elevated caspase-3 activity and MMP depolarization, which also proved the radiosensitivity of BMSCs in radiation models in vitro^2–4,7^.

IR commonly poses direct and indirect threats to cellular ROS induction. Direct IR damage to cells leads to dysfunctional organelle states and perturbed signaling networks, which induces intracellular ROS production^26^. Mitochondria are deemed the source of intracellular energy and host most intracellular ROS generation. Electron leakage occurs in the ETC when oxidative phosphorylation and ATP production occur in mitochondrial domains, leading to the accumulation of H_2_O_2,_ O_2_^−^ and other oxidative species, also termed mitochondrial-dependent ROS generation^26,27^. In the current study, both total ROS and mitochondrial ROS were significantly increased when BMSCs were exposed to 6 Gy of IR, and this excessive ROS production was blocked by pretreatment with the antioxidant NAC or MitoQ.

Increasing evidence supports that excess mitochondrial oxidation contributes to ischemia/reperfusion injury, activates apoptosis signaling, and promotes cell death, bone marrow injury, inflammation, etc^4,9,11,15,16,28,29^. Under normal regulation, apoptosis is a highly regulated and conserved process that is necessary for maintaining intracellular homeostasis. However, the dysfunctional state of apoptosis has been associated with cell death, fibrosis, atrophy and biological inhibition. Under oxidative stress, the permeability of the inner mitochondrial membrane (IMM) is increased, which allows the permeation of dangerous signals and results in perturbed oxidative phosphorylation and dysfunctionality^16^. All of these factors collapse the MMP, activate pro-apoptotic genes, such as cytochrome C, caspase-9, caspase-3, Bax, Apaf-1, Bid and Bad, and downregulate pro-survival genes, such as Bcl-2 family members and survivin, thus triggering the mitochondrial apoptosis pathway^30,31^. We also found that IR-induced mitochondrial ROS was significantly associated with cellular apoptosis, verified by elevated caspase-3 activity and MMP depolarization. The acquisition of radioresistance was attainable by pretreatment with NAC via decreasing ROS derived from mitochondrial following IR exposure.

Autophagy, a defensive mechanism, is a conserved catabolic process that maintains intracellular homeostasis by delivering cellular constituents to lysosomes, eliminating dangerous signals and recycling cytoplasmic content. Autophagic activation was characterized by the formation of autophagosomes and the elevated expression of autophagy genes (ATGs), LC-3, Beclin1, p62, etc^32^. The effects of radiation on autophagy and apoptosis have been thoroughly discussed. Clinical exposure, especially IR, is one of the most important exogenous stimulants that induces autophagy among various cell types^17,32–35^. However, the mechanism underlying IR-induced autophagy has not been fully elucidated. Numerous studies using autophagy to alter cancer cell radiosensitivity have been performed to improve therapeutic efficiency^33,34,36^. Due to its cytoprotective function, inhibiting autophagy facilitates IR-induced apoptosis or cell fate decisions in tumor cells. This protective role of IR-induced autophagy, in turn, can be applied to increase the radioresistance of BMSCs, thus alleviating IR damage and maintaining cellular viability^19^. We also proved that autophagy was activated in irradiated BMSCs, which showed attenuated apoptosis caused by IR exposure. In addition, IR-induced autophagy helped counteract the accumulation of IR-induced mitochondrial ROS, which may partially explain how autophagy exerts cytoprotective effects^19,37,38^.

Radiation-induced expression changes in miR-22 have been widely reported, with upregulation being observed in mouse skin, HEK293T, U87MG and TK6 cells and downregulation being reported in cancer stem cells^20,39–42^. We are the first to elucidate miR-22 upregulation in a time-dependent manner in BMSCs following IR. As a multi-functional molecule, miR-22 participates in the regulation of cell survival, drug-induced apoptosis, ROS generation, autophagy, tumor biology, neuroprotection and ischemia/reperfusion injuries^13,15,20,22,43,44^. However, the biological role of miR-22 in radiation response has not been clearly elucidated. Here, we found that miR-22 was a key positive regulator of IR-induced mitochondrial ROS and cellular apoptosis. Although IR activated autophagy and upregulated miR-22 expression, miR-22 overexpression via the transfection of mimics significantly inhibited this biological response, indicating that miR-22 plays a negative role in autophagy via a feedback loop and the existence of other pathways regulating autophagy formation, which was in line with findings postulated by Sciarretta et al.^44^. As mentioned above, we believe that IR-induced BMSC apoptosis is partially regulated by the miR-22-mediated accumulation of mitochondrial ROS and autophagy inhibition.

Redd1, initially known as a stress response gene, is induced by radiation, DNA damage and other dangerous signals and is well known for its regulatory role in autophagy via the suppression of mTORC1 activity^45,46^. Through microRNA-arrays, Li XH et al. validated the miR-30c in CD34 + cells and human fetal osteoblast cells, which can negatively regulate the expression of Redd1, thus mediating different biological events^25^. Similarly, using a dual luciferase assay, Redd1 was identified as a direct target gene of miR-22. We are the first to elucidate the miR-22/Redd1 pathway in the regulation of radiation response. In tumorigenesis, Peter H et al. found that endogenous Redd1 accumulated in mitochondria wherein ROS were generated and served as a regulator of its metabolism. Furthermore, Redd1^−/−^ cells exhibited significantly elevated mitochondrial ROS and mTORC1 activity, as shown by the stabilization of HIF-1, as well as impaired mitochondrial function^45,47^. In current investigation, we also found the negative regulation of Redd1 on mTORC1 activity, which indicated that the participation of Redd1 in cellular autophagy may be mTORC1-dependent. Redd1^+/+^ fibroblasts and tissues exhibited increased resistance to IR, alleviating the apoptotic ratio and promoting recovery from DNA damage^45^. Redd1 deficiency impeded ROS-induced autophagy and disturbed mitochondrial metabolism in an osteoarthritis model^23^. In the current investigation, Redd1 overexpression significantly attenuated the accumulation of mitochondrial ROS and enhanced cellular autophagy, thus protecting BMSCs from IR injury. Furthermore, the upregulation of Redd1 blocked miR-22-mediated mitochondrial ROS and autophagy inhibition, thus reversing IR-induced cell damage.

In conclusion, the present study demonstrates that Redd1 targets the regulation of miR-22-mediated mitochondrial ROS and cellular autophagy during IR-induced apoptosis. This finding further elucidates the biological mechanisms and a novel molecular mechanism underlying ionizing radiation-induced BMSC injury. Further studies should focus on the correlation between ROS and autophagy and the detailed mechanisms underlying Redd1, especially the specific mechanism that Redd1 mediated on autophagy.

## Supplementary information

Figure S1

Figure S2

Figure S3

Figure S4

Figure S5

Figure S6

Figure S7

Figure S8

Supplemental figure legends
