# Supplementary figures and images for: Regulatory roles of miR-22/Redd1-mediated mitochondrial ROS and cellular autophagy in ionizing radiation-induced BMSC injury
Source: Cell Death Dis. 2019 Mar 7;10(3):227. doi: 10.1038/s41419-019-1373-z (PMC6405932; doi:10.1038/s41419-019-1373-z)

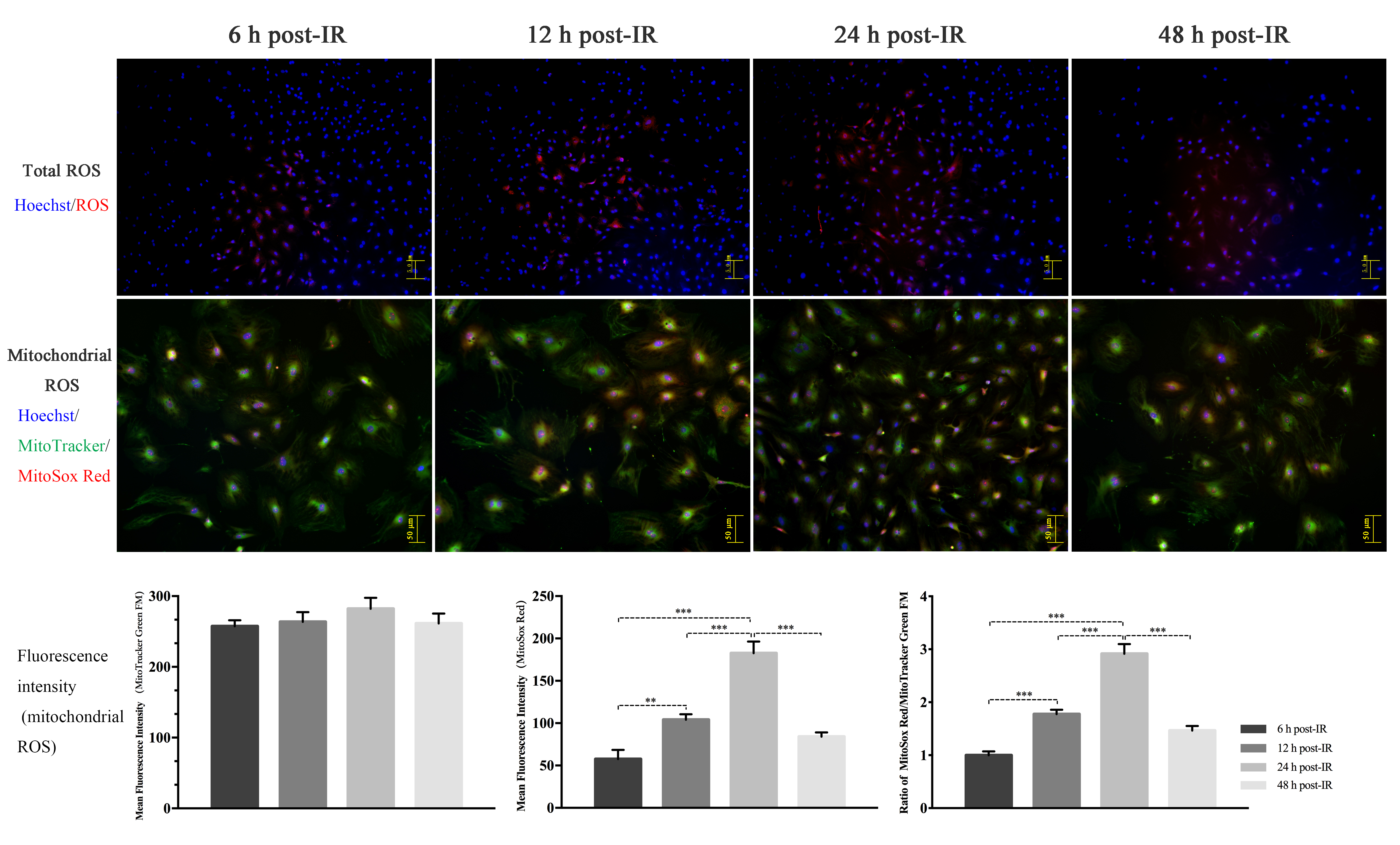

Supplement: Supplementary file 1 — Figure S1 [file 41419_2019_1373_MOESM1_ESM.tif]

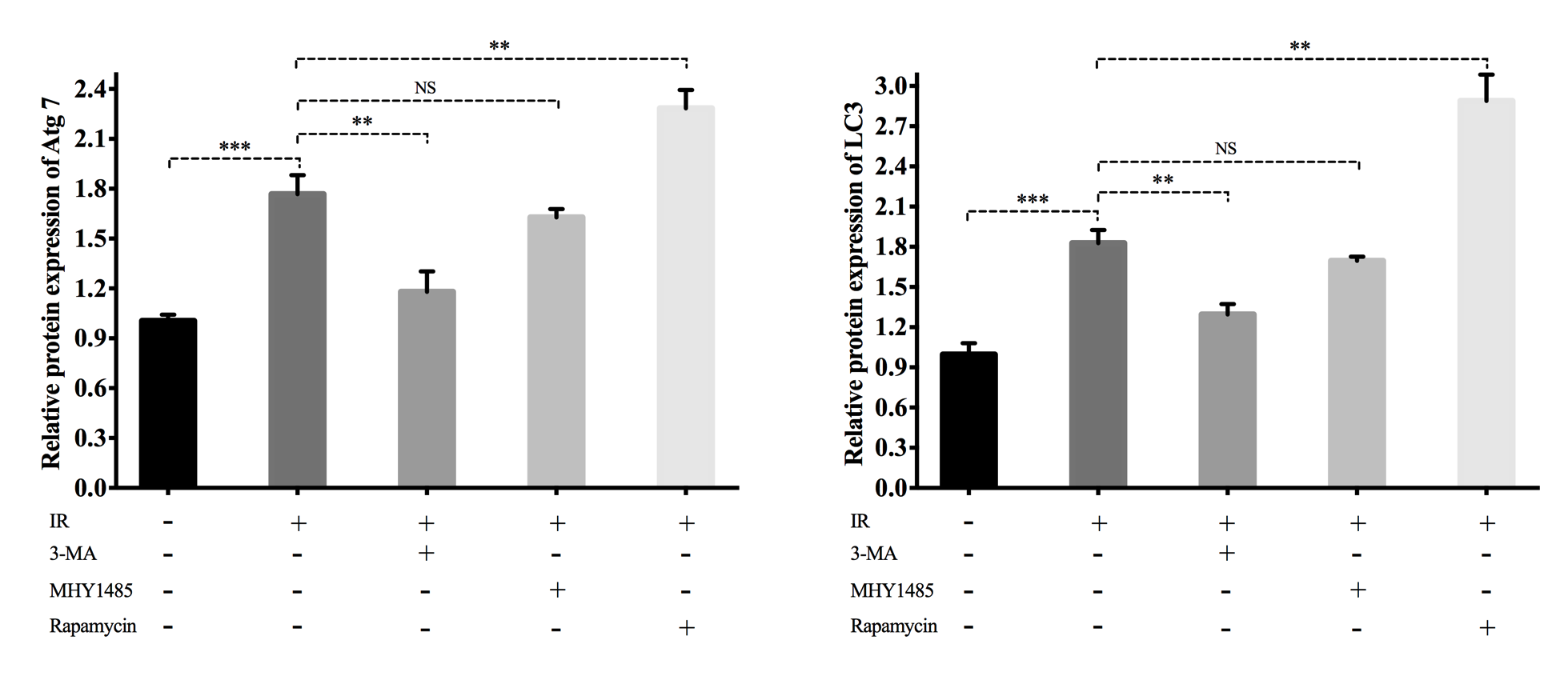

Supplement: Supplementary file 2 — Figure S2 [file 41419_2019_1373_MOESM2_ESM.tif]

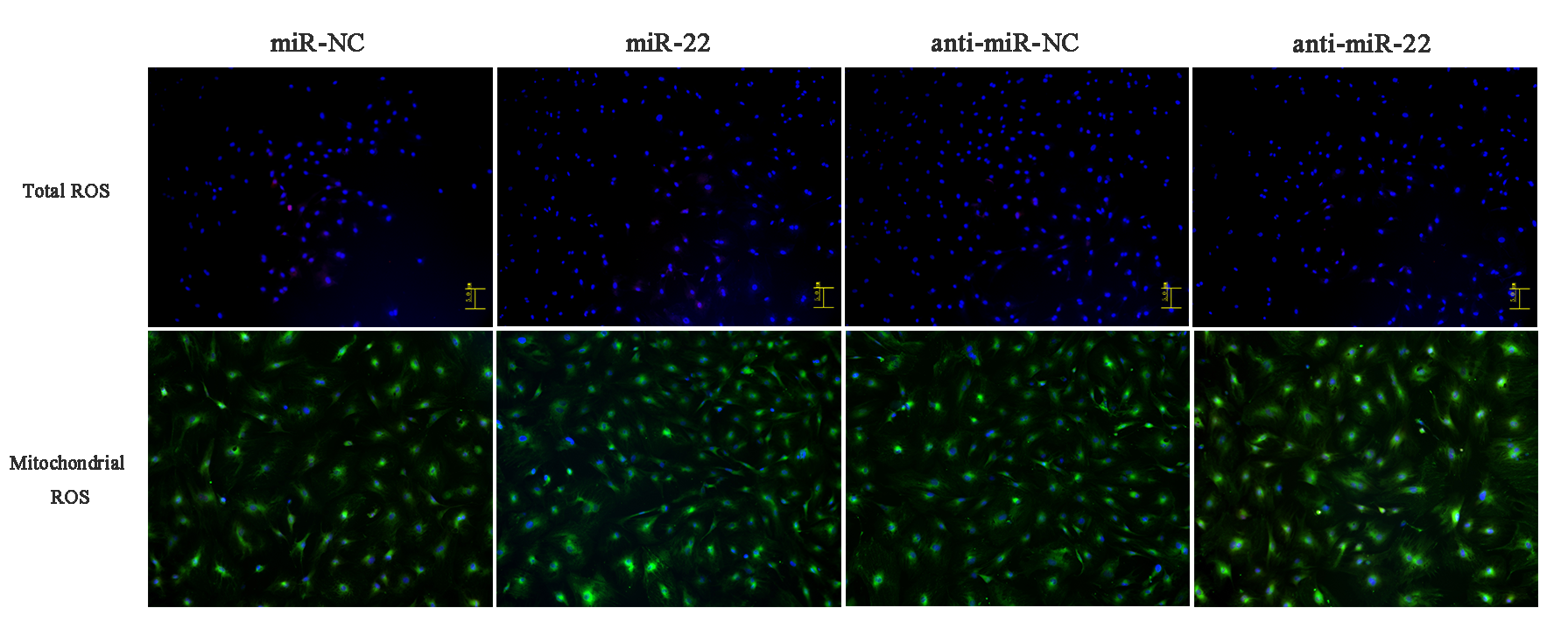

Supplement: Supplementary file 3 — Figure S3 [file 41419_2019_1373_MOESM3_ESM.tif]

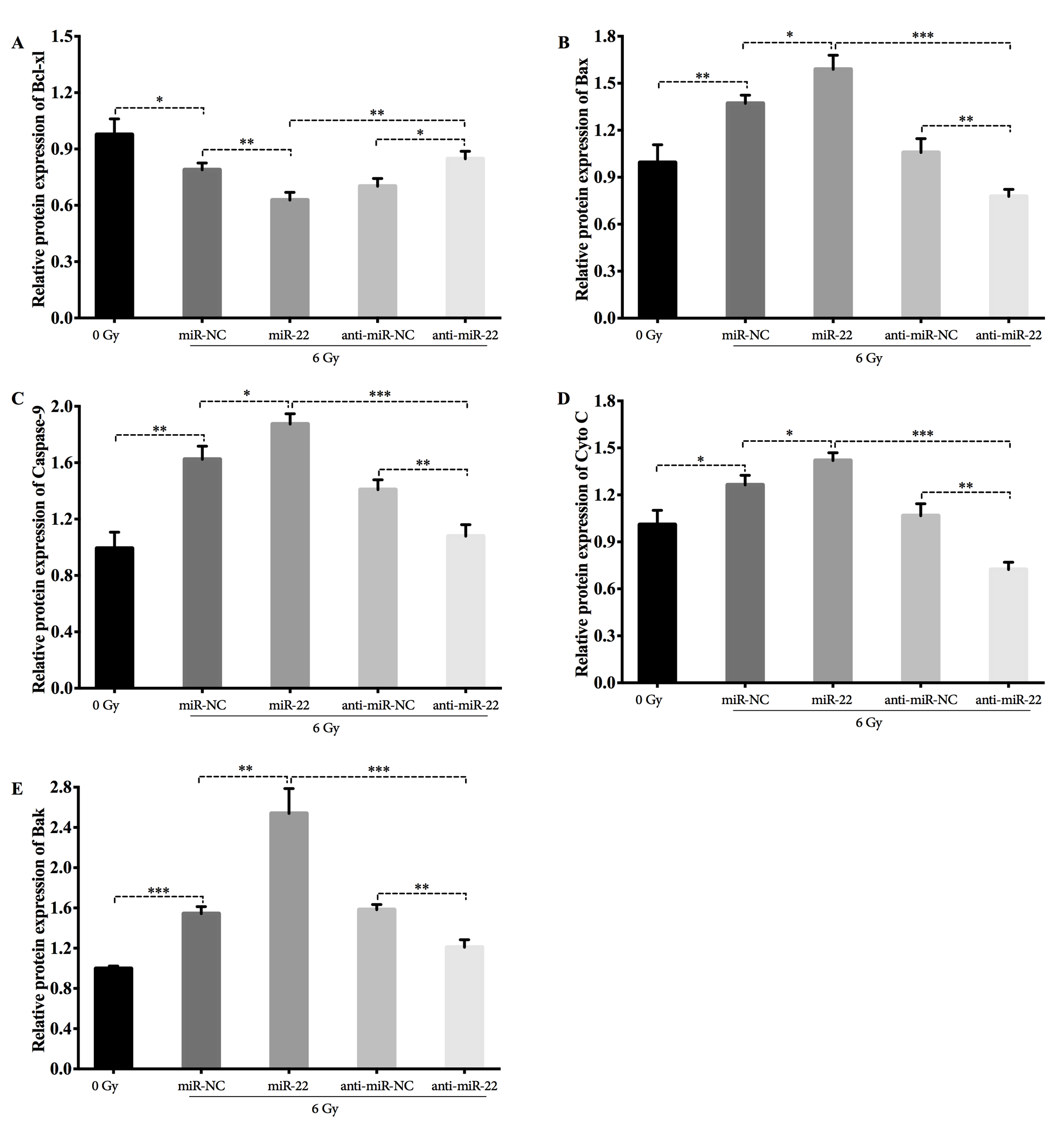

Supplement: Supplementary file 4 — Figure S4 [file 41419_2019_1373_MOESM4_ESM.tif]

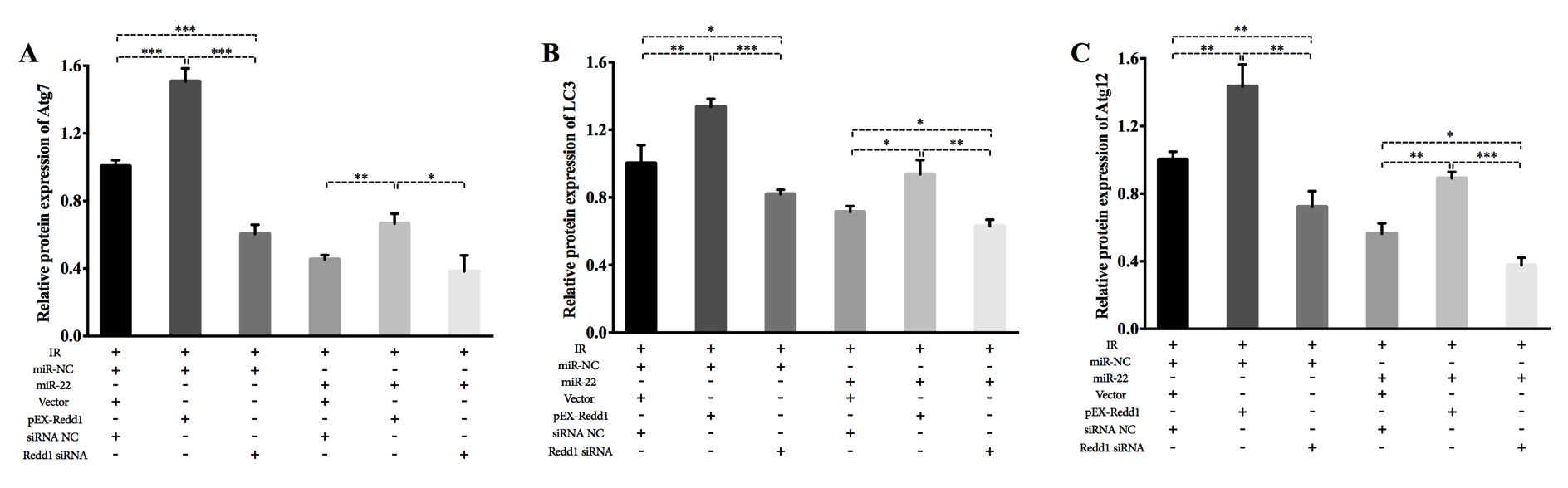

Supplement: Supplementary file 5 — Figure S5 [file 41419_2019_1373_MOESM5_ESM.tif]

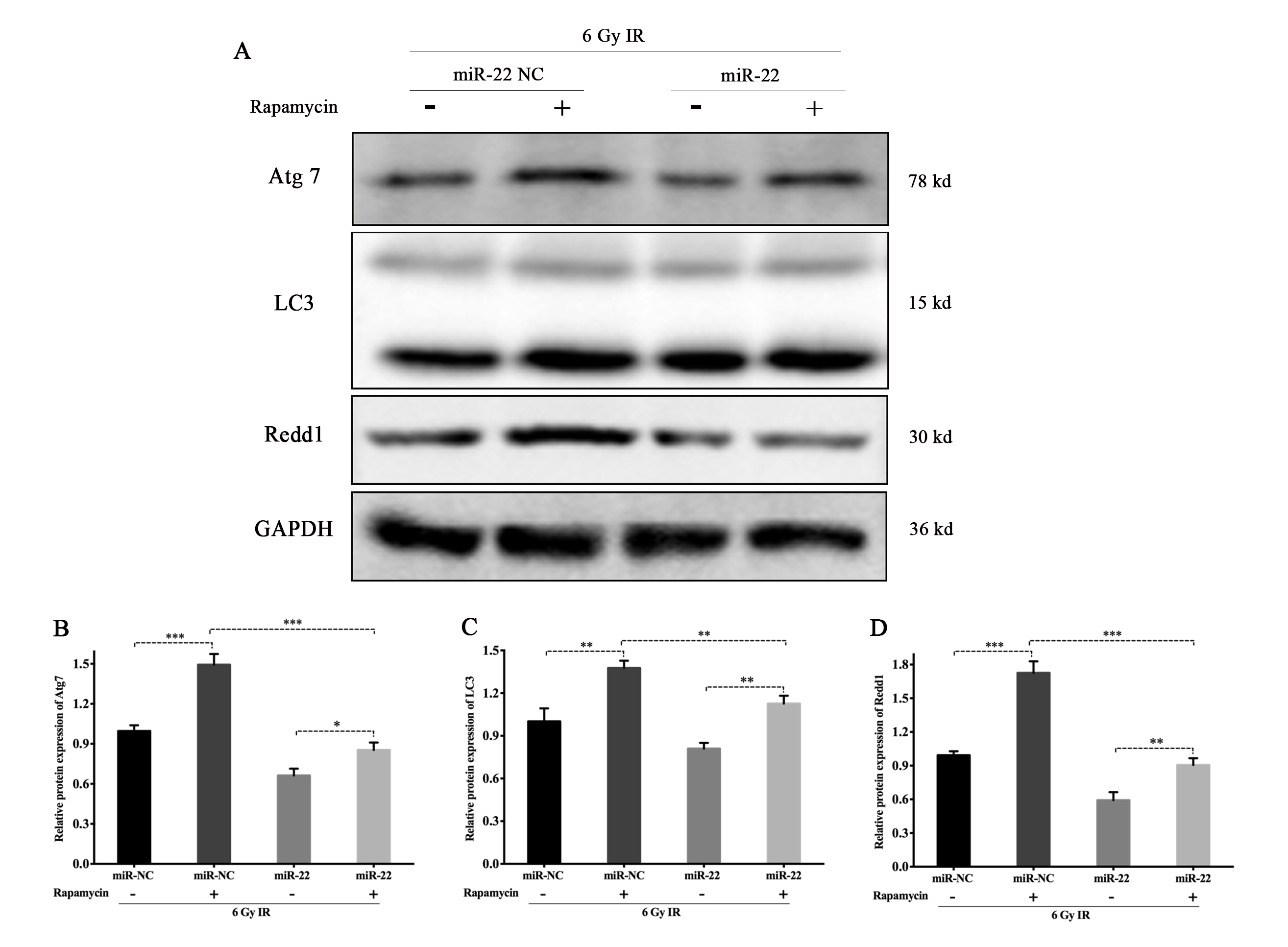

Supplement: Supplementary file 6 — Figure S6 [file 41419_2019_1373_MOESM6_ESM.tif]

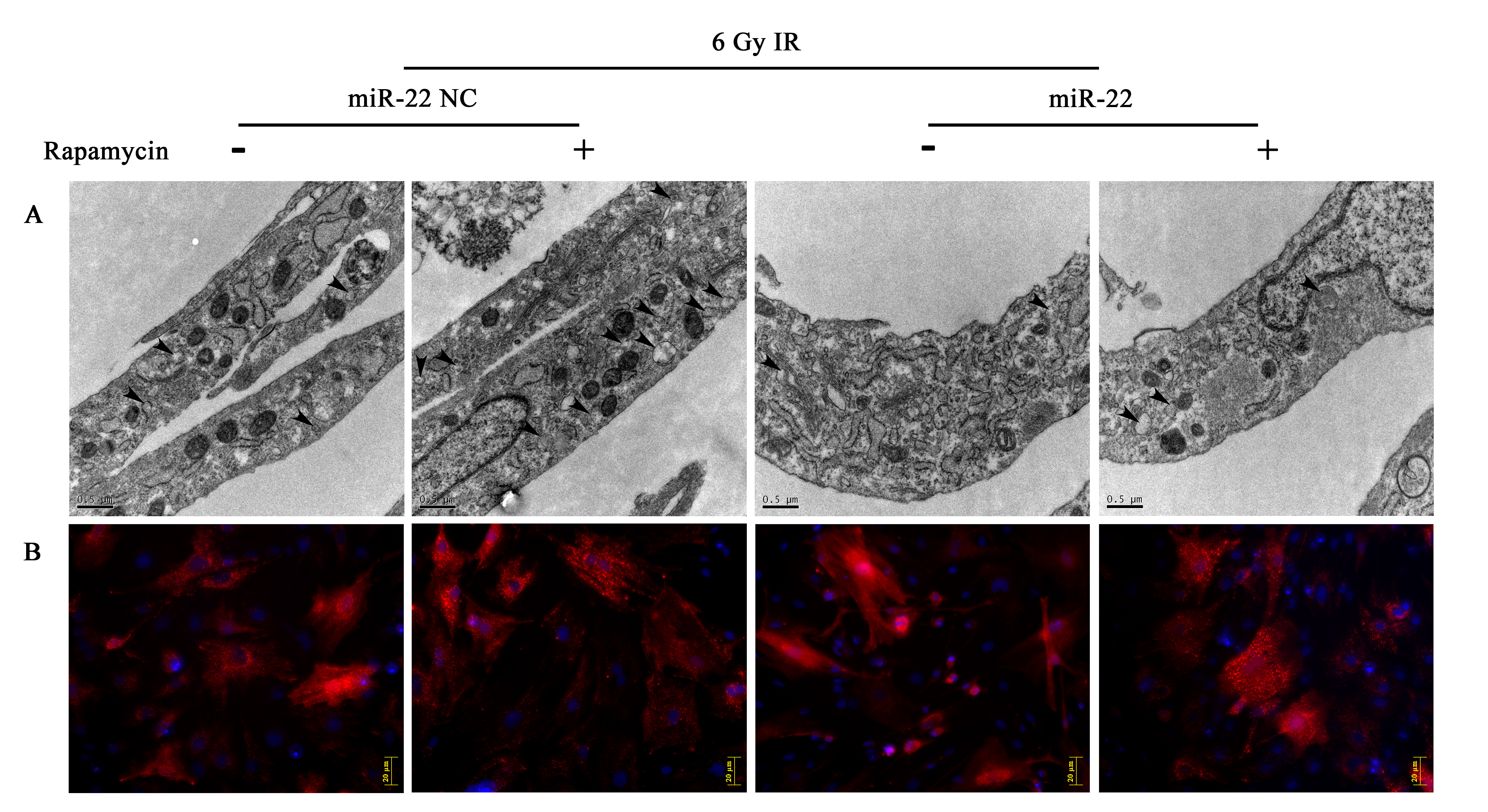

Supplement: Supplementary file 7 — Figure S7 [file 41419_2019_1373_MOESM7_ESM.tif]

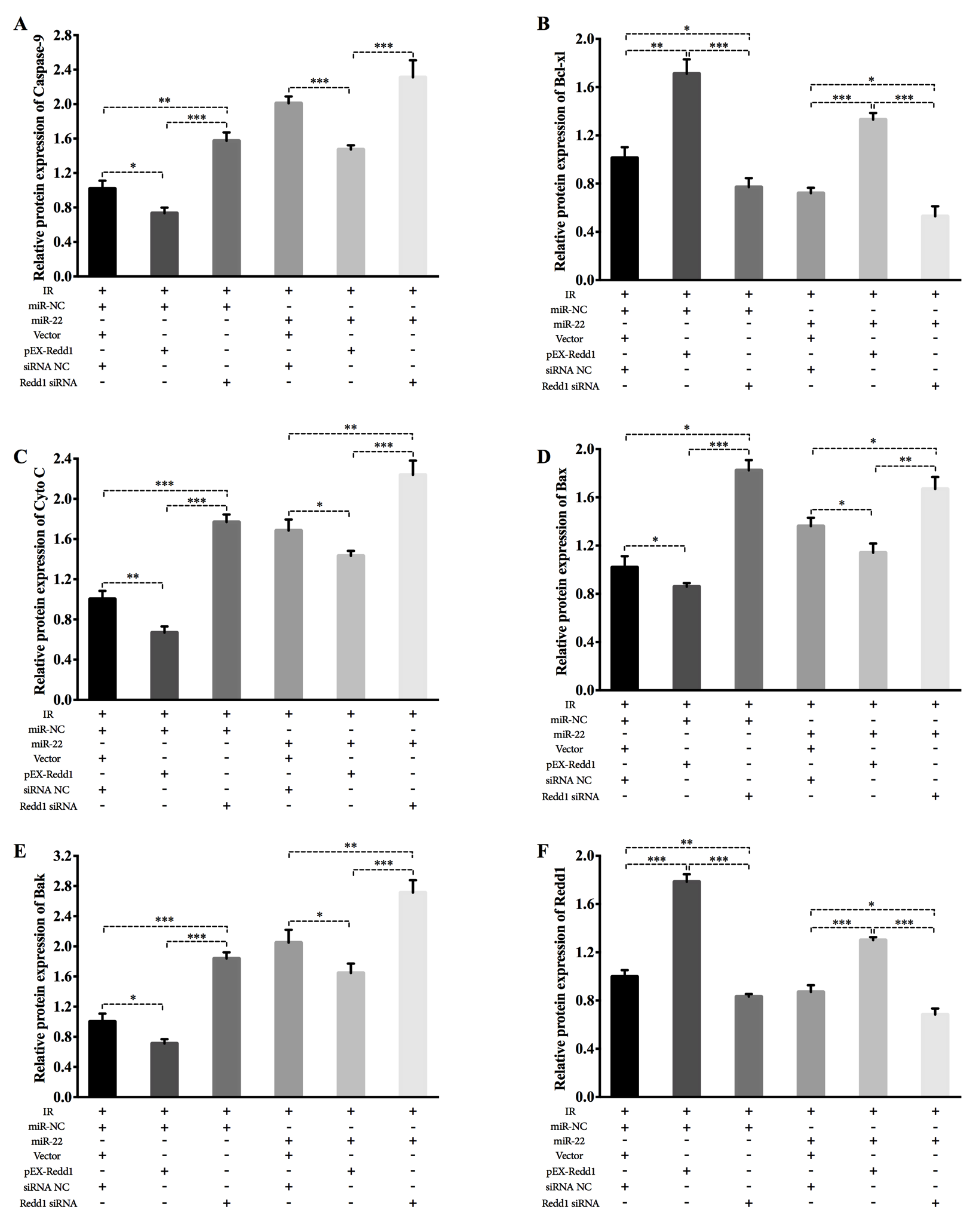

Supplement: Supplementary file 8 — Figure S8 [file 41419_2019_1373_MOESM8_ESM.tif]
