# Supplementary material for: Regulatory roles of miR-22/Redd1-mediated mitochondrial ROS and cellular autophagy in ionizing radiation-induced BMSC injury
Source: Cell Death Dis. 2019 Mar 7;10(3):227. doi: 10.1038/s41419-019-1373-z (PMC6405932; doi:10.1038/s41419-019-1373-z)
Supplement: Supplementary file 9 — Supplemental figure legends [file 41419_2019_1373_MOESM9_ESM.doc]

**Supplementary figure legends**

Figure S1 The total and mitochondrial ROS in irradiated rBMSCs at 6, 12, 24 and 48 h post IR.

**Figure S2** Relative expression of autophagic related protein (Atg7, LC3) under IR, 3-MA, MHY1485 and rapamycin treatment. (** *p*≤0.01; *** *p*≤0.001; NS: no siginificance)

**Figure S3** The influence of miR-22 transfection alone (without IR exposure) on intracellular total and mitochondrial ROS. A. intracellular total ROS; B. mitochondrial ROS.

**Figure S4** Relative expression of mitochondrial-dependented apoptotic related protein (Caspase-9, Bcl-xl, Bak, Bax and Cyto C) under miR-22 modification and subsequent 6 Gy IR exposure. (* *p*≤0.05; ** *p*≤0.01; *** *p*≤0.001)

**Figure S5** Relative expression of autophagic related protein (Atg7, LC3, Atg12) under miR-22/Redd1 modification and subsequent 6 Gy IR exposure. (* *p*≤0.05; ** *p*≤0.01; *** *p*≤0.001)

Figure S6 The mediated role of rapamycin on Redd1 expression and miR-22 mediated suppression of autophagy. A. protein expression of autophagic related markers (Atg7, LC3) under IR, miR-22 and rapamycin treatment; B-D. relative expression of autophagic related protein (Atg7, LC3). (* *p*≤0.05; ** *p*≤0.01; *** *p*≤0.001)

Figure S7 The mediated role of rapamycin on miR-22 mediated suppression of autophagy. A.（T.E.M analysis）Black arrow represents the autophagosome; B. Ad-mCherry-LC3B analysis.

**Figure S8** Relative expression of mitochondrial-dependented apoptotic related protein (Caspase-9, Bcl-xl, Bak, Bax and Cyto C) under miR-22/Redd1 modification and subsequent 6 Gy IR exposure. (* *p*≤0.05; ** *p*≤0.01; *** *p*≤0.001)
